# A Multifaceted Study of *Scedosporium boydii* Cell Wall Changes during Germination and Identification of GPI-Anchored Proteins

**DOI:** 10.1371/journal.pone.0128680

**Published:** 2015-06-03

**Authors:** Sarah Ghamrawi, Amandine Gastebois, Agata Zykwinska, Patrick Vandeputte, Agnès Marot, Guillaume Mabilleau, Stéphane Cuenot, Jean-Philippe Bouchara

**Affiliations:** 1 L'UNAM Université, Université d'Angers, Groupe d'Etude des Interactions Hôte-Pathogène, EA 3142, Angers, France; 2 L'UNAM Université, Université de Nantes, Institut des Matériaux Jean Rouxel, Nantes, France; 3 Laboratoire de Parasitologie-Mycologie, Centre Hospitalier Universitaire, Angers, France; 4 L'UNAM Université, Service Commun d'Imageries et Analyses microscopiques, Angers, France; Hans-Knoell-Institute (HKI), GERMANY

## Abstract

*Scedosporium boydii* is a pathogenic filamentous fungus that causes a wide range of human infections, notably respiratory infections in patients with cystic fibrosis. The development of new therapeutic strategies targeting *S*. *boydii* necessitates a better understanding of the physiology of this fungus and the identification of new molecular targets. In this work, we studied the conidium-to-germ tube transition using a variety of techniques including scanning and transmission electron microscopy, atomic force microscopy, two-phase partitioning, microelectrophoresis and cationized ferritin labeling, chemical force spectroscopy, lectin labeling, and nanoLC-MS/MS for cell wall GPI-anchored protein analysis. We demonstrated that the cell wall undergoes structural changes with germination accompanied with a lower hydrophobicity, electrostatic charge and binding capacity to cationized ferritin. Changes during germination also included a higher accessibility of some cell wall polysaccharides to lectins and less CH_3_/CH_3_ interactions (hydrophobic adhesion forces mainly due to glycoproteins). We also extracted and identified 20 GPI-anchored proteins from the cell wall of *S*. *boydii*, among which one was detected only in the conidial wall extract and 12 only in the mycelial wall extract. The identified sequences belonged to protein families involved in virulence in other fungi like Gelp/Gasp, Crhp, Bglp/Bgtp families and a superoxide dismutase. These results highlighted the cell wall remodeling during germination in *S*. *boydii* with the identification of a substantial number of cell wall GPI-anchored conidial or hyphal specific proteins, which provides a basis to investigate the role of these molecules in the host-pathogen interaction and fungal virulence.

## Introduction

*Scedosporium* species are filamentous fungi commonly isolated from polluted soils and water, but paradoxically infrequent in the air and indoor environment [1–5]. Recent taxonomic studies revealed that *Pseudallescheria boydii*, now called *Scedosporium boydii* [6], and *Scedosporium apiospermum* which was initially considered its asexual state, are two distinct species. These two along with three other closely related species, *S*. *dehoogii*, *S*. *aurantiacum* and *S*. *minutisporum*, constitute the *Scedosporium apiospermum* species complex [7–9].

Depending on the portal of entry and the patient’s immune status, these usually saprophytic fungi may be at the origin of a wide variety of human infections, ranging from localized infections subsequent to traumatic inoculation of fungal elements as in subcutaneous mycetomas, to disseminated infections in immunocompromised individuals [1, 10].

In the past two decades, these fungi gained worldwide recognition as the second most frequently isolated filamentous fungi in the airways of patients with cystic fibrosis, the most common autosomal recessive disease in Caucasian populations [11–14]. In CF patients, these fungi usually colonize the respiratory tract and may contribute to the progressive deterioration of the lung function as suggested by recent works on other fungal species like *Candida albicans* and *Aspergillus fumigatus* [15–17]. In addition, this chronic colonization of the airways constitutes a risk factor for severe and often fatal disseminated infections in patients undergoing lung transplantation, which remains the ultimate treatment in CF [18, 19]. Until now, the diagnosis of *Scedosporium* infections remains challenging mainly because of the similarities of clinical features and histopathology with other relatively common hyaline hyphomycetes like *Aspergillus* or *Fusarium* species. Add to this, *Scedosporium* species exhibit low susceptibility to amphotericin B and current triazole drugs as well as primary resistance to echinocandins [20, 21]. There is, therefore, an urgent need for a better understanding of the fungal biology in order to define new therapeutic strategies.

One of the most attractive targets for the development of new antifungal agents is the cell wall, mainly because of the uniqueness of many of its components with respect to mammalian cells [22]. The cell wall plays a critical role during morphogenesis and fungal growth since it changes accordingly to fit survival needs [23]. It protects the fungus from a wide range of environmental stresses such as desiccation, osmotic stresses and temperature variations. In pathogenic fungi it also provides the means to sustain fungal presence inside the human host by allowing adherence to the host tissues and evasion from the host immune response.

In *S*. *boydii*, which is the most prevalent species within the *S*. *apiospermum* species complex in CF [24], the conidial and mycelial cell walls were shown to contain *N*- and *O*-linked peptidorhamnomannans (PRM) having a branched structure of α-Rha*p*-(1→3)-α-Rha*p*- side chain epitope linked (1→3) to a (1→6)-linked α-Man*p* core [25]. Unlike α-glucans, isolated from both conidial and hyphal cell walls of *S*. *boydii*, glucosylceramides could only be obtained from mycelial samples [26, 27]. However a more recent study again showed that glucosylceramides were also detectable on the conidial surface of the fungus [28].

We previously demonstrated that the conidial cell wall of *S*. *boydii* contains dihydroxynaphtalene (DHN)-melanin and that the cell wall content in melanin and mannose-containing glycoconjugates increases during maturation of conidia along with the cell surface physical properties [29]. Here, we tracked the cell wall modifications during the germination process using various approaches, including investigation, at the molecular level, of glycosylphosphatidylinositol (GPI)-anchored proteins in conidial and hyphal walls as these integral cell wall proteins (CWPs) play a major role in normal morphology and virulence in other fungal models [30–32].

## Materials and Methods

### Strain and culture conditions

The fungal strain *S*. *boydii* IHEM 15155 (formerly *Pseudallescheria boydii*) was used throughout this study [29]. It was maintained on yeast extract-peptone-dextrose (YPD; 0.5% w/v yeast extract, 2% w/v glucose, 1% w/v peptone, 0.05% w/v chloramphenicol) agar plates.

Cultures were incubated for 7 days at 37°C, then conidia were harvested by flooding the agar surface with sterile Milli-Q water and filtered through a 20-μm pore size nylon filter. Conidia were washed twice, pelleted at 5000 X *g* for 5 min at 4°C, resuspended in 10 ml sterile water and finally counted with a hemocytometer.

### Kinetics of germination

To study the kinetics of germination, three conditions were tested: the effect of age of cultures, culture medium and incubation temperature. First, cultures on YPD agar medium were incubated at 37°C for 5, 9 and 14 days, afterwards conidia were isolated and resuspended in YPD liquid medium (20 ml per Petri dish) at a concentration of 2 x 10^6^ conidia/ml and kept at 37°C. To study the effects of culture medium, the same settings were applied except that the fungus was cultivated on Malt (1.5% w/v malt extract, 0.05% w/v chloramphenicol) or YPD agar and then conidia were resuspended in Malt or YPD liquid media which were incubated at 37°C. Finally, for the incubation temperature, conidia taken from cultures on YPD agar at 37°C were resuspended in YPD liquid medium which was incubated at 20°C, 25°C, or 37°C. In all cases, germination in liquid media was monitored over 8 h and five pictures were taken every 2 h, the presence of mycelia was also checked after 16 h. The percentage of germination was determined after counting at least 100 cells from each picture.

### Scanning and transmission electron microscopy

Resting conidia or germ tubes cultured in YPD medium for 6, 8, 10 or 24 hours were washed twice in Milli-Q water and once in 0.1 M cacodylate buffer, and then incubated in the fixative solution (2.5% (w/v) glutaraldehyde, 2% (w/v) paraformaldehyde, 0.1 M cacodylate buffer) for 24 h at room temperature under vacuum. After washing with cacodylate buffer, samples were incubated for 24 h in 2% KMnO_4_ in cacodylate buffer at 4°C, washed and post-fixed for 2 h at room temperature in 2% osmium tetroxide. Then samples were washed in Milli-Q water and finally dehydrated through a series of ethanol-water solutions (50, 70, 95% ethanol, 2 x 30 min each) and then 100% ethanol (3 x 20 min).

For scanning electron microscopy (SEM), samples underwent two baths of graded ethanol-hexamethyldisilazane (HDMS) solutions (50/50, then 25/70 proportions, 45 min each) followed by immersion in pure HMDS baths (3 x 45 min). Processed samples were mounted on aluminium stubs, coated with carbon, and stored in a desiccator until studied. Observations were made on a JSM 6301F scanning electron microscope (Jeol, Paris, France) operating at 3 kV and equipped with digital imaging.

For transmission electron microscopy (TEM), ethanol was replaced by propylene oxide (3 x 20 min) and samples were impregnated overnight in a propylene oxide-Epon mixture (1:1 v/v) and then in pure Epon for 16 h and 8 h. After polymerization (24 h at 37°C, 24 h at 45°C and then 48 h at 60°C), thin sections were directly examined on a JEM-1400 transmission electron microscope (Jeol, Paris, France; 120 kV) except for life cycle studies of *S*. *boydii* where thin sections were contrasted with uranyl acetate and lead citrate prior examination.

### Ferritin labeling

Cationized ferritin is a positively charged ligand that allows visualization of anionic sites at the cell surface under physiological pH and ionic strength [33]. *Scedosporium boydii* germ tubes were examined by TEM after labeling with cationized ferritin, and controls consisted in incubation of fungal elements with native ferritin (lacking a positive charge) and in pretreatment of germ tubes with neuraminidase (type X) in order to remove sialic acids (all products purchased from Sigma-Aldrich, St Quentin Fallaviers, France). To do this, *S*. *boydii* germ tubes were washed 3 times with Milli-Q water, centrifuged and then incubated with cationized or native ferritin (1 mg/ml in phosphate buffered saline 150 mM) for 1 h at room temperature with agitation. To remove sialic acids, cells were first incubated with neuraminidase (1 U/ml in 0.1 M acetate buffer pH 5, supplemented with 40 mM CaCl_2_) for 30 min at room temperature with shaking, washed twice and then incubated with cationized ferritin as described above. Cells in the three conditions were finally washed twice in Milli-Q water and treated as described earlier for transmission electron microscopy.

### Cell surface charge and hydrophobicity measurement

Cell surface charge and hydrophobicity were evaluated by microelectrophoresis and two-phase partitioning as described previously [29]. For evaluation of the cell surface charge, resting or germinating conidia (10^6^ cells) were washed and resuspended in Milli-Q water containing 1 mM NaCl, then their electrophoretic mobility was measured at 25°C using Zetasizer Nano ZS (Malvern Instruments Ltd, Malvern, UK). The cell surface hydrophobicity (CSH) was determined using the water/hexadecane system. Briefly, germ tube and conidial suspensions in PBS were topped (except for control samples) with hexadecane, vortexed and then allowed to stand at room temperature for 3 min; then 1 ml of the aqueous phase (bottom) was transferred into a new tube and vortexed and finally the aqueous phase was transferred to a microplate and read at an optical density (OD) of 405 nm. The percentage difference in optical density readings between test samples and controls was considered as the hydrophobic index. All experiments were performed in triplicate.

### Lectin labeling

Germ tubes were washed with Tris buffer (0.5 mM Tris, 100 mM NaCl, 1 mM CaCl_2_, 1 mM MgCl_2_, pH 7.0) and then incubated for 30 min at 37°C under continuous rotatory mixing with gold-conjugated concanavalin A (Con A-gold 5 nm from Biovalley, Marne la Vallée, France; 1:50 dilution in Tris buffer) or with FITC-conjugated Con A, peanut agglutinin (PNA) or wheat germ agglutinin (WGA) at a final concentration of 100 μg/ml (all fluorescent lectins from Sigma-Aldrich) [29]. Control samples consisted in incubation of fungal elements together with the lectins and a large excess (0.2 M) of the lectin-specific carbohydrates (α-methyl D mannopyranoside for Con A, N-acetyl glucosamine for WGA, and galactose for PNA) added immediately before lectins. Finally, samples were washed three times in Tris buffer, and observed under fluorescence microscope (Leica DMR, Leipzig, Germany) for FITC-conjugated lectins or processed as described earlier for TEM without contrasting with uranyl acetate and lead citrate.

### Chemical force spectroscopy (CFS) measurements

The surface of *S*. *boydii* resting or germinated conidia was imaged using a NanoWizard atomic force microscope (JPK Instruments AG, Berlin, Germany) operating in intermittent contact mode under ambient conditions. A standard rectangular cantilever (Nanosensors NCL-W) was used for imaging, with a free resonance frequency of 165 kHz and a typical spring constant of about 40 N/m. The radius curvature of the tip was ~10 nm. The detailed analysis of chemical force spectroscopy images was performed using JPK Data Processing software (JPK Instruments AG). Hydrophilic and hydrophobic adhesions were obtained in ultrapure water from force-distance curves measured on the surface of both conidia and germ tubes using functionalized cantilevers. Gold-coated cantilevers (Olympus, Hambourg, Germany) with spring constants of 0.01 N/m were immersed either in 1 mM solutions of 1-dodecanethiol or in 11-mercapto-1-undecanol (Sigma-Aldrich) in ethanol for 14 h and then rinsed with ethanol prior their use. From force-curve measurements (2048 measurements), the mean hydrophilic and hydrophobic adhesions were extracted from gaussian fits performed on the histograms. Before probing the conidial or germ tube surface, the cantilevers functionality was tested by measuring their adhesion to hydrophobic or hydrophilic flat surfaces.

### Protein extraction, identification and analysis

#### Protein extraction

Extraction was performed according to Damveld *et al*. [34] with modifications. Frozen conidia or germ tubes were ground with a mortar and pestle in liquid nitrogen, then crushed in a cell homogenizer (Braun Melsungen model MSK, Melsungen, Germany) for 1.5 min for conidia or 1 min for germ tubes (150 mg dry material) under a current of CO_2_ cooling in the presence of a protease inhibitor cocktail (15 ml; 1X in Milli-Q water, cOmplete, EDTA-free, Roche, Meylan, France) and a mix of 1 mm and 0.25 mm diameter glass beads. Glass beads were removed by filtration through 41-μm-pore size sterile nylon filters and cell breakage was confirmed by phase-contrast microscopy (> 95%). Suspensions were centrifuged at 13500 X *g* for 10 min at 4°C. After lyophilization, cell debris (500–900 mg) were washed 5 times with 50 mM Tris-HCl buffer pH 7.8 (25 μl per mg dry weight) and pelleted at 18000 X *g* for 10 min at 4°C. Cytosolic contaminants, membrane proteins and disulfide-linked cell wall proteins were removed by boiling five times (2 min each) with SDS-extraction buffer (50 mM Tris-HCl pH 7.8, 2% w/v SDS, 0.1 M Na-EDTA, and 1.6 μl β-mercaptoethanol; 25 μl per mg dry weight). Then cell wall debris were washed six times with Milli-Q water, lyophilized and weighed. To extract cell wall GPI-anchored proteins, freeze-dried cell wall debris were incubated with hydrofluoric acid (HF)-pyridine (10 μl per mg dry weight) for 3 h at 0°C [35]. Then the suspension was centrifuged at 18000 X *g* for 10 min at 4°C and proteins were precipitated from the supernatant by the addition of 9 volumes of 100% methanol-Tris buffer (100% v/v methanol, 50 mM Tris-HCl pH 7.8) followed by incubation for 2 h at 0°C. After centrifugation, the pellet was washed twice with 90% methanol-Tris buffer (90% v/v methanol, 50 mM Tris-HCl pH 7.8) and lyophilized. The resulting extracts represented 10.0% and 10.2% of the initial dry weight of cell wall debris after removal of cytosolic contaminants in conidia and germ tubes, respectively. Finally, proteins were deglycosylated with peptide-N-glycosidase F (PNGase F glycerol free, New England Biolabs, Evry, France) according to the manufacturer’s recommendation. Deglycosylation was performed in glass vials by the addition of 5000 units PNGase F to 50–100 mg protein extract, followed by incubation for 3 h at 37°C. Proteins were precipitated and washed as described earlier using the methanol-Tris buffers and finally lyophilized.

#### Trypsin digestion

Lyophilized samples were suspended in 50 mM ammonium bicarbonate and incubated with 7.2 mM dithiothreitol (DTT) during 15 min at 37°C. They were next incubated with 13.5 mM iodoacetamide during 15 min at room temperature in the dark. Samples were then digested overnight with 4 ng/μl of sequencing grade modified trypsin (Promega, Madison, WI, USA) at 37°C.

#### Nano liquid chromatography-tandem mass spectrometry (nanoLC-MS/MS)

Tryptic peptides were separated on a nanoflow high-performance liquid chromatography (nano-HPLC) system (Dionex, Villebon sur Yvette, France; LC Packings Ultimate 3000) connected to a hybrid LTQ-OrbiTrap XL (Thermo Fisher Scientific, Villebon sur Yvette, France) equipped with a nanoelectrospray ion source (New Objective, Wil, Switzerland). They were first concentrated onto a trapping precolumn (5 mm x 300 μm i.d., 300 Å pore size, Pepmap C18, 5 μm) and then separated and eluted by reverse-phase using an analytical column (15 cm x 300 μm i.d., 300 Å pore size, Pepmap C18, 5 μm; Dionex, LC Packings) with a 140 min, 2–90% acetonitrile gradient in 0.05% formic acid at a flow rate of 0.25 μl/min. The mass spectrometer was operated in its data-dependent mode by automatically switching between full survey scan MS and consecutive MS/MS acquisition. Survey full scan MS spectra (mass range 400–2000) were acquired in the OrbiTrap section of the instrument with a resolution of R = 60000 at m/z 400. The ten most intense peptide ions in each survey scan with an intensity above 2000 counts and a charge state = 2 were sequentially isolated and fragmented in the linear ion trap by collision induced dissociation (CID). For OrbiTrap measurements, an external calibration was used before each injection series ensuring an overall error mass accuracy below 5 ppm for the detected peptides. MS data were saved in RAW file format (Thermo Fisher Scientific) using XCalibur 2.0.7 with tune 2.5.5 SP1.

#### Protein identification

The Proteome Discoverer 1.2 software was used to submit MS/MS data to the translated genome of *S*. *apiospermum* IHEM 14462 [36] completed with *Sus scrofa* trypsin and *Elizabethkingia miricola* PNGase F (10829 sequences) using the Mascot search engine (Mascot server v2.2; http://www.matrixscience.com). Parameters were set as follows: trypsin as enzyme with one allowed miscleavage, carbamidomethylation of cysteins as fixed modification and methionine oxidation as variable modifications. Mass tolerance for MS and MS/MS was set at 10 ppm and 0.5 Dalton, respectively. Identified rank 1 peptides were filtered based on the Mascot score to obtain a false discovery rate of 1%.

#### Protein analysis

Identified protein sequences were analyzed for the presence of GPI anchor using Big PI fungal predictor (http://mendel.imp.ac.at/gpi/fungi_server.html), signal peptide using Signal P (http://www.cbs.dtu.dk/services/SignalP/), transmembrane helices using TMHMM server (http://www.cbs.dtu.dk/services/TMHMM/), N- and O-glycosylation sites using NetNGlyc and NetOGlyc services (http://www.cbs.dtu.dk/services/), pI using ProtParam (http://web.expasy.org/protparam/) and grand average of hydropathy values (GRAVY) to evaluate the hydrophilic or hydrophobic character of a protein along its amino acid sequence (http://www.bioinformatics.org/sms2/protein_gravy.html). For pipeline filtering of proteins the new web tool proFasta was also used (http://www.bioinformatics.nl/tools/profasta/). Functional domain analysis was performed using Interpro (http://www.ebi.ac.uk/interpro/). Protein sequence similarities were searched by using Blastp in NCBI website (http://blast.ncbi.nlm.nih.gov) against all the non-redundant protein sequences and then against species-specific databases *Aspergillus fumigatus*, *Neurospora crassa*, *Magnaporthe oryzae* and *Saccharomyces cerevisiae*. Specific websites were also used to perform Blastp analysis against *Candida albicans* (http://www.candidagenome.org), *Colletotrichum graminicola* and *Colletotrichum higginsianum* genomes (http://www.broadinstitute.org/annotation/genome/colletotrichum_group/Blast.html).

### Statistical analysis

For studies of the kinetics of germination, two-way analysis of variance (ANOVA) was used with the Bonferroni post-hoc test. For cell surface charge and hydrophobicity studies, results were analyzed using the Student *t*-test. *P*-values less than 0.05 were considered significant.

## Results

### Germination of *S*. *boydii*

**Fig 1:**
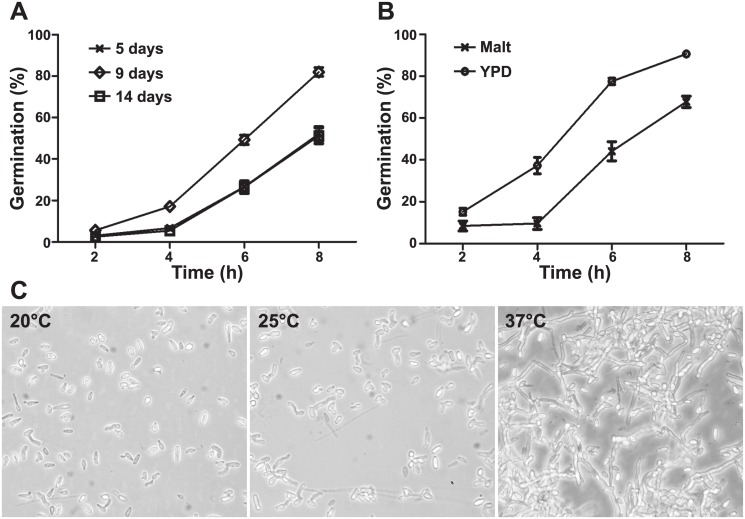
Kinetics of germination of *S*. *boydii* in various conditions. **(A**) Conidia isolated from 5-, 9- and 14-day-old cultures on yeast peptone dextrose (YPD) agar were incubated in YPD liquid medium over 8 h at 37°C. (**B**) Conidia isolated from 9-day-old cultures on Malt or YPD agar were incubated in Malt or YPD liquid media over 8 h at 37°C. (**C**) Conidia isolated from 9-day-old cultures on YPD agar were incubated in YPD liquid medium for 16 h at 20°C, 25°C or 37°C (200X).

In order to define the best conditions for germination of *S*. *boydii*, three different parameters were first investigated: the age of culture, the culture medium and incubation temperature. Tracking germination during 8 h showed significant differences (*P* < 0.01 at 4 h and *P* < 0.001 at 6 and 8 h) in the percentages of germination between conidia recovered from 5-, 9-, and 14-day old cultures (Fig 1A). Germination started after 4 h of incubation and conidia recovered from 9-day-old cultures showed highest rates of germination as well as more homogeneity in terms of length of germ tubes. Prolonging the duration of cultivation to 14 days resulted in a decrease in the germination rate possibly because conidia started entering a state of long dormancy or because they were dying. After 16 h of incubation all cultures showed mycelial agglomerations and the percentage of germination could no more be determined.

Germination was also affected by the culture medium. Higher germination rates were observed for conidia recovered from 9-day-old cultures and incubated in YPD liquid medium compared to malt liquid medium (Fig 1B; *P* < 0.001). Finally, incubation temperature greatly affected the kinetics of germination since the number and length of germ tubes progressively increased with the increase in incubation temperature from 20°C to 25°C and 37°C, with the presence of long branched intermixed hyphae after 16 h of incubation at 37°C (Fig 1C). Therefore germination was performed by incubation of conidia from 9-day old cultures in YPD liquid medium at 37°C for all subsequent experiments.

**Fig 2:**
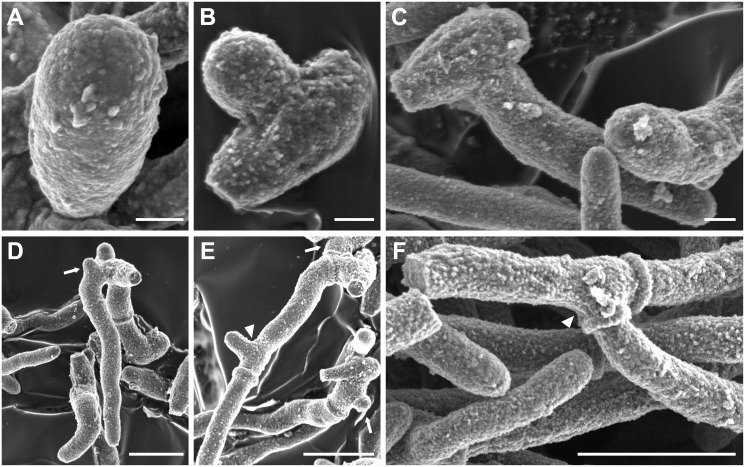
The life cycle of *S*. *boydii* under scanning electron microscopy. After release, conidia (**A**) germinate (**B**) and the hyphal part of germ tubes elongates (**C**) until a first branch emerges near the mother cell (**D**). Both hyphae grow and more branching sites appear on filaments at the subapical region of the articles (**E**) until the mother cell can no more be distinguished (**F**). Arrows indicate sites of first branching, and later branching are indicated by arrowheads. All cultures were performed in YPD broth with incubation at 37°C for 6h (**B**), 8h (**C**), 10 h (**D** and **E**) or 24 h (**F**). Bars: 1 μm in A, B and C; 0.5 μm in D; and 5 μm in E and F.

The process of germination in *S*. *boydii* starts by the protrusion of a germ tube from the mother cell without significant differences in the conidial size before and after initiation of the germination process. As illustrated in Fig 2A, resting conidia measured 5.31 ± 0.92 x 2.74 ± 0.57 μm (length x width; 7 conidia studied), a size which remains essentially the same after germination (Fig 2B; 4.79 ± 0.36 x 2.13 ± 0.34 μm; length x width; 7 conidia measured). Most germination events occurred laterally rather than along the axis of the mother cell: Among 111 germinated cells only 29 cells (26.1%) germinated along the axis whereas the rest of conidia (73.9%) germinated laterally as shown in Fig 2B and 2C. With the progression of germination, branching occurred and the first branching site was seen very close to the mother cell (Fig 2D and 2E, arrow). Finally, more branching appeared along the filaments, at the subapical region of the articles (Fig 2E and 2F, arrowheads), and filaments elongated until the mother cell was no more distinguished.

**Fig 3:**
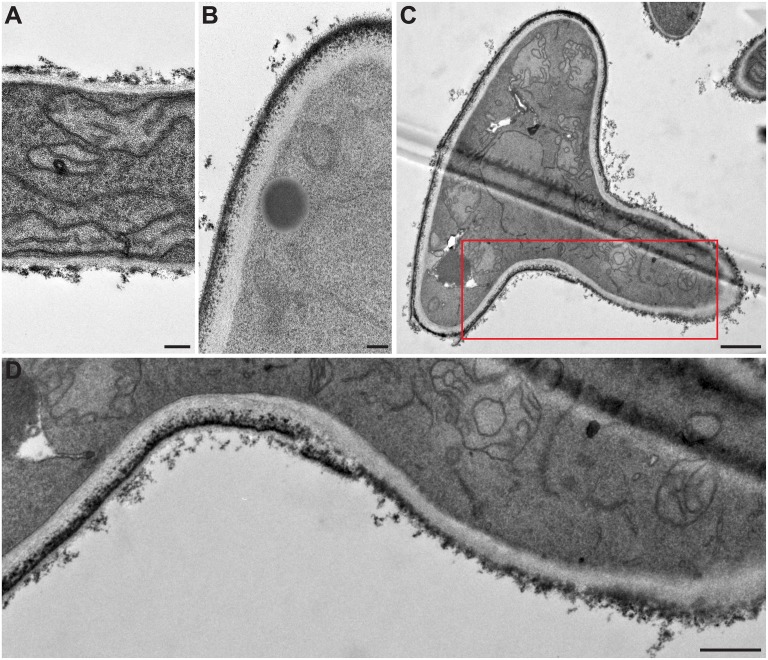
Cell wall modifications during germination of *S*. *boydii* under transmission electron microscopy. **(A**) hyphal cell wall; **(B**) conidial cell wall; and **(C**) cell wall of a germinating conidium. (**D)** Enlarged part of (**C)** highlighting the cell wall structural modifications during germination, particularly the electron dense outer layer being less continuous at the surface of the hyphal part of germ tubes. Bars: 0.2 μm in A and B; 1 μm in C; and 0.5 μm in D.

TEM examination of the different morphological stages of the fungus showed important ultrastructural changes in the cell wall with germination (Fig 3). The fungal cell wall appeared to be composed of two layers, a large electron transparent inner layer and a thinner electron dense outer layer. Comparing the cell wall of hyphae (Fig 3A) to that of conidia (Fig 3B) showed a homogeneous thickness of the outer cell wall layer in resting conidia and mother cells of germ tubes, whereas its thickness greatly varied in hyphae. The outer cell wall layer of the filament was thin or even absent in some restricted areas. Of note, the cell wall surface of the mother cell seemed to be covered by a thin electron dense layer which was less continuous at the surface of the hyphal part of germ tubes, possibly in relation with the necessary plasticity of the cell wall for hyphal elongation (Fig 3C and 3D).

### Changes in cell surface physical properties during germination

**Fig 4:**
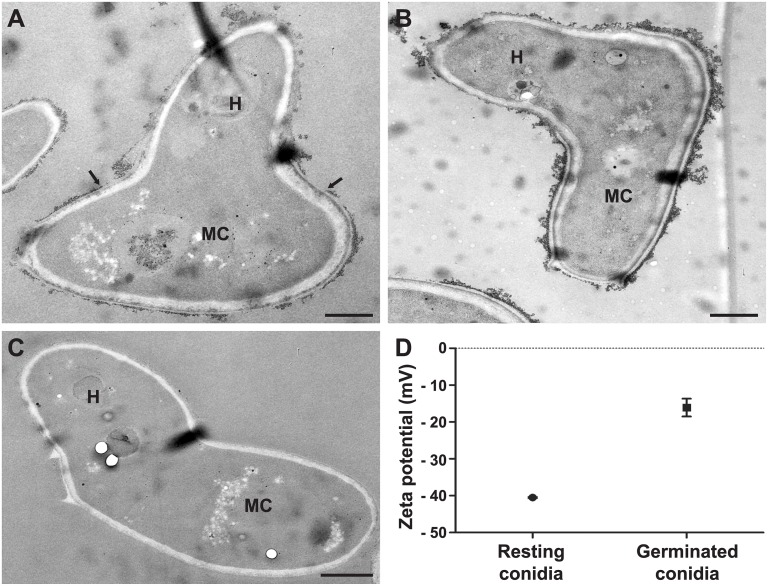
Surface charge modifications during germination of *S*. *boydii* by ferritin labeling and zeta potential measurements. TEM images of germ tubes labeled with cationized ferritin (**A**), germ tubes treated with neuraminidase prior cationized ferritin labeling (**B**) or germ tubes incubated with native ferritin (**C**). (**D**) Comparison of the surface electrostatic charge of resting and germinated conidia calculated from the electrophoretic mobility of 10 000 cells using Zetasizer Nano ZS (*P* = 0.0005). **H**: hyphal part of germ tube; **MC**: mother cell of germ tube. Bars: 1 μm.

The differences in the biochemical composition of the cell wall between conidia and hyphae reflected by the ultrastructural changes were also revealed by modifications of the surface physical properties as can be seen in Fig 4A. TEM after cationized ferritin labeling showed a greater density of negatively charged areas at the surface of mother cells of germ tubes compared to their hyphal part. Neuraminidase treatment before incubation with cationized ferritin did not reduce the binding of cationized ferritin, suggesting that the surface electronegative charge was not connected to sialic acids (Fig 4B). The use of native ferritin confirmed that binding of cationized ferritin was related to its electrostatic charge and not to the ferritin molecule itself (Fig 4C). Zeta potential measurements also provided evidence for the difference in the surface electronegative charge between resting and germinated conidia as seen in Fig 4D (- 40.50 mV *vs*.—16.10 mV; *P* = 0.0005).

Similarly, a significant difference in the cellular hydrophobicity between resting and germinated conidia was revealed by two-phase partitioning using the water/hexadecane system. The mean percentage of resting conidia excluded from the aqueous medium, which reflects the number of hydrophobic cells within the whole population, was 37.8 ± 0.7 compared to 2.9 ± 1.3 for germinated conidia (*P* < 0.0001).

### Changes in the cell surface composition during germination

#### Lectin binding to cell wall carbohydates

**Fig 5:**
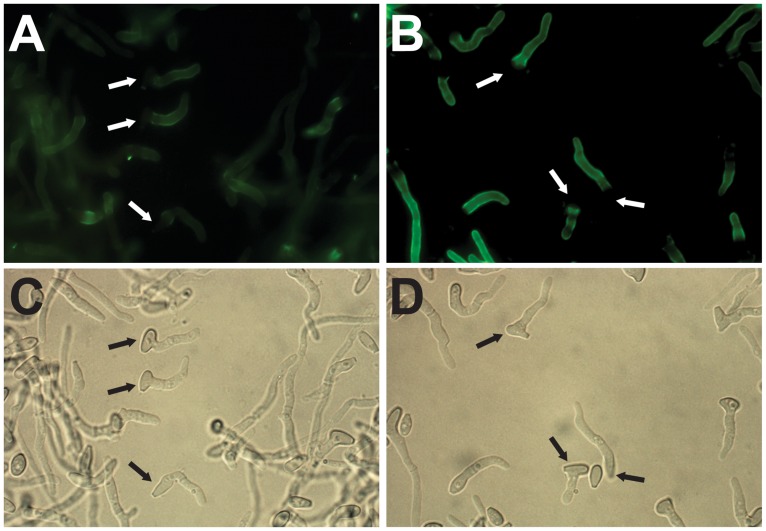
Fluorescence labeling of *S*. *boydii* surface carbohydrates with FITC-conjugated lectins. Germ tubes after labeling with concanavalin A (**A** and **C**) or wheat germ agglutinin (**B** and **D**) lectins. The same fields are presented under fluorescence (**A** and **B**) and phase contrast microscopy (**C** and **D**) respectively. Arrows indicate mother cells.

**Fig 6:**
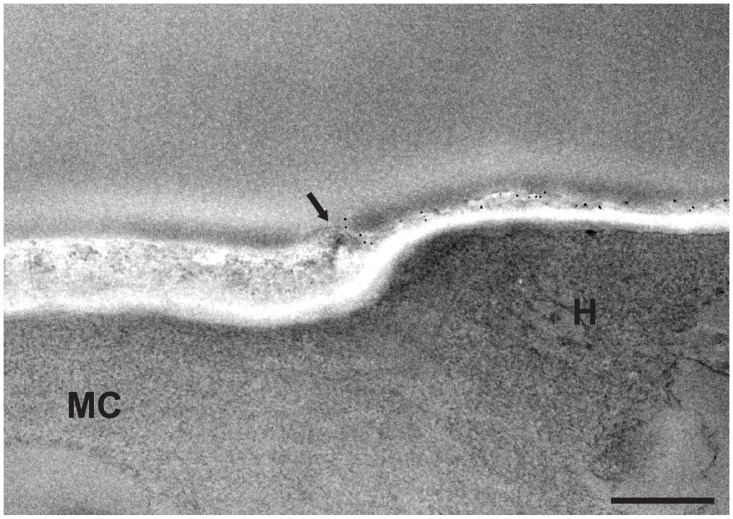
Gold labeling of cell wall mannan groups in *S*. *boydii* germ tubes. Germ tubes labeled with gold-conjugated concanavalin A (Con A; 5-nm gold particles) showing higher affinity of gold particles to the hyphal part (H) of germ tubes compared to the mother cell (MC) under transmission electron microscopy. Arrow indicates the limit of the outer cell wall layer of the mother cell. Bar: 0.5 μm.

The accessibility of cell wall carbohydrates to mannose-binding, chitin-binding [37] and galactose-binding [38] lectins (Con A, WGA and PNA, respectively) was investigated during germination of *S*. *boydii*. While most of the mother cells were not labeled with Con A, as previously shown for resting conidia [29], the hyphal part of almost all germ tubes was intensely labeled (Figs 5A, 5C and 6), suggesting unmasking of the mannose-containing glycoconjugates by the loss of the surface electron dense film seen on the mother cells. A similar binding pattern was also seen for WGA (Fig 5B and 5D), whereas no fluorescence was observed after incubation with PNA either on the mother cell or the hyphal part of germ tubes.

#### Detection of hydrophobic/hydrophilic adhesions at high spatial resolution in conidia and hyphae

**Fig 7:**
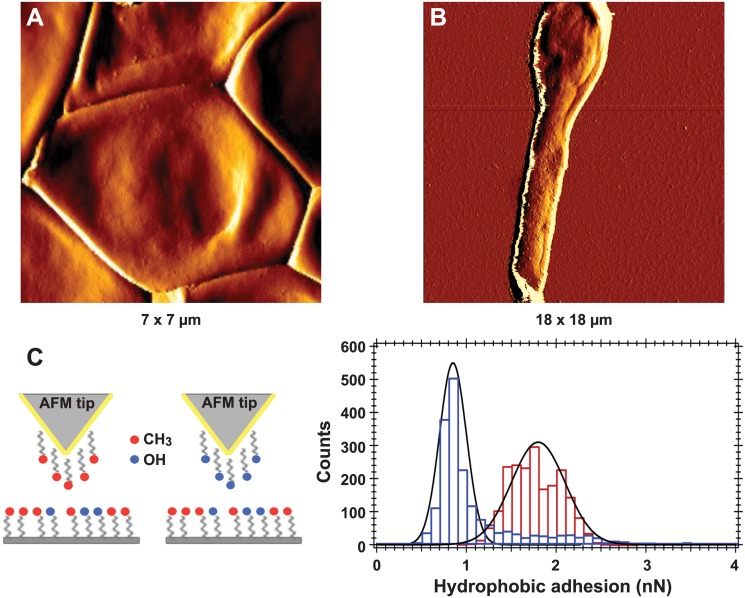
High resolution imaging and chemical force spectroscopy analysis of *S*. *boydii* resting conidia and germ tubes. AFM amplitude images of a resting (**A**) or germinated (**B**) *S*. *boydii* conidium. (**C) Left**, scheme for chemical functionalization of AFM tips. Gold-coated tips were modified with CH_3_-terminated alkanethiols or OH-terminated alkanethiols. (**C**) **Right**, histograms of hydrophobic adhesion forces measured on the surface of a resting conidium (1.8 ± 0.3 nN, in red) and the hyphal part of a germinated conidium (0.85 ± 0.15 nN, in blue).

AFM images presented on Fig 7A and 7B revealed a smooth conidial cell wall surface, devoid of any peculiar organization, conversely to that of *A*. *fumigatus* conidia which is totally covered by rodlets [39]. To investigate the chemical nature of *S*. *boydii* cell surface, non-specific force-curves were measured with OH- or CH_3_-modified tips (Fig 7C) to determine the distribution of hydrophobic or hydrophilic components at the surface of individual resting conidia and its evolution with germination.

The use of CH_3_-modified probes (to measure hydrophobic adhesion forces) revealed the presence of hydrophobic components, such as glycoproteins. Force-curves recorded on the surface of resting conidia with CH_3_ tips showed large adhesion forces of 1.8 ± 0.3 nN, whereas a lower value of 0.85 ± 0.15 nN was obtained on the hyphal surface (Fig 7C), which was in agreement with the diminished cell surface hydrophobicity of germ tubes compared to resting conidia measured by two-phase partitioning. The values for CH_3_/CH_3_ interactions (hydrophobic adhesions) measured on the trunk and the apex of the germ tube were similar (0.8 to 0.9 nN) suggesting that the same hydrophobic components (glycoprotein) covered the whole hyphal surface.

The use of OH-modified probes revealed hydrophilic adhesion forces mainly due to the presence of polysaccharides. On the contrary to results of CH3/CH3 interaction measurements, no significant difference in OH/OH interactions (hydrophilic adhesions) was observed between resting (1.2 ± 0.4 nN) and germinated conidia (1.0 ± 0.2 nN). Also, the same OH/OH adhesion values (1.0 nN) were calculated for the trunk and apex of the hyphal part of germ tubes.

### Characteristics of the identified cell wall proteins

GPI-anchored proteins selectively released with HF-pyridine from SDS buffer washed cell wall fragments, were first analysed on SDS-PAGE. Nevertheless, extracted proteins were revealed only by silver staining. Due to the small amount of proteins, analysis of glycosylation was not performed and attention was focused on the identification of the extracted proteins by nanoLC-MS/MS. Consequently deglycosylated HF-extracts were analysed with nanoLC-MS/MS which detected two hundred and fifty four proteins from both the conidial and germ tube extracts taken together. *In silico* analysis of these proteins identified 32 proteins with a signal peptide among which 20 proteins had a GPI-anchor following analysis with BigPI software (Table 1 and S1 Table). Seven out of the 20 GPI-anchored proteins were detected in both conidial and germ tube extracts, 12 only in the germ tube extract and one only in the conidial extract. All identified GPI-anchored proteins were predicted to be glycosylated (Table 2), and all but one (glycine-rich protein, accession number KEZ42341.1) had no transmembrane helices after excluding 45 N-terminal and 35 C-terminal amino acids. One transmembrane helix was found for the glycine-rich protein (accession number KEZ42341.1). All the twenty proteins had a pI < 5 except for the glycine-rich protein whose pI was 6.89. To analyze the serine (S)—threonine (T) content of our proteins, we first considered the overall S/T content of proteins (results indicated in Table 2). A further analysis was performed with proFasta using additional filters (S/T content > 10%, *Start position of scan* 26 and *End position of scan* -26) to exclude the S/T content of N- and C-terminal signal sequences. This analysis showed that all identified GPI-anchored proteins had an S/T content higher than 10%.

**Table 1:** GPI-anchored proteins identified in conidial and/or germ tube extracts.

Protein (accession number)	Protein family	Function	Ref.	Fungal extract^*a*^	Sequence^*b*^
Glucan endo-1,3-β-D-glucosidase (KEZ41172.1)	Bglp/Bgtp (Bgt2p) GH 17	Cell wall remodeling: branching β-(1,3) glucan through the formation of β-(1,6) linkage	[73, 74]	RC	AAQGLDGTNGAFNSAR
				GT	ISPTGIANKEFAGANPDTLVGYIK
					EFAGANPDTLVGYIK
					AAQGLDGTNGAFNSAR
					SQSDFEAEFKAAQGLDGTNGAFNSAR
CFEM protein (KEZ46909.1)	CFEM domain	Fungus-host interaction (plant infection, heme- uptake, biofilm structure)	[77–80]	RC	AGEFGcQSTDVAcLcR
					SRDFVYGIR
				GT	AGEFGcQSTDVAcLcR
					SRDFVYGIR
					DFVYGIR
CFEM protein (KEZ46627.1)	CFEM domain	Fungus-host interaction (plant infection, heme- uptake, biofilm structure)	[77–80]	RC	QGDWYcGcQPDNmSK
				GT	QGDWYcGcQPDNMSK
					IQGAATNcVIEAcGGAAGALAVITEVQGIcEEALK
CFEM protein (KEZ44163.1)	CFEM domain	Fungus-host interaction (plant infection, heme- uptake, biofilm structure)	[77–80]	RC	IPEcANScVTQATSGNK
				GT	IPEcANScVTQATSGNK
					IAGcNQGDIK
GDSL_like lipase (KEZ43142.1) ^*c*^		Unknown function in fungi		RC	SQKVVLVDFR
				GT	MAALLFDGINNAASR
Glycine-rich protein (KEZ42341.1)		Unknown function in fungi		RC	GGSSSSSSSSSRPGSPGFAGSGAPR
				GT	GGSSSSSSSSSRPGSPGFAGSGAPR
Unknown (KEZ44256.1)		Unknown function		RC	NTcEALcPGAAK
				GT	YYSASLYSFVcQEAFK
					NTcEALcPGAAK
CRH1_transglycosylase (KEZ42985.1)	Crhp (Crh1p) GH16	Cell wall remodeling: cross link chitin to β-(1,3) and β-(1,6) glucan	[68–71]	GT	GAVFSIANEK
					LGSWVAGR
					GGKTYPQTPMQVK
					TYPQTPMQVK
					DcPADPAIGGDFTVDFTK
GH17 family protein (SAPIO_CDS10506)	Bglp/Bgtp (Bgt2p), GH17	Cell wall remodeling: branching β-(1,3) glucan through the formation of β-(1,6) linkage	[73, 74]	GT	DSNPDNKMQFAITK
					NAPGKFNAVR
					AGIGADPSVLVGFIGDYR
Unknown (KEZ45212.1)		Unknown function		GT	AcGATDYDcQcAAQQAISTcYNNcPGDSRK
					AcGATDYDcQcAAQQAISTcYNNcPGDSR
1,3-β-glucanosyltransferase gel4 (KEZ46619.1)	Gelp/Gasp, (AfGel5p) GH72	Cell wall remodeling: elongation of β(1–3)glucan	[30], [71–73]	GT	GIAYQQNTGAAGAGVQDAK
					FFYENGTQFYIK
Unknown (KEZ45428.1)		Unknown function		GT	QGLLGVVANAEDGVLYAcSQVK
CFEM protein (KEZ43031.1)	CFEM domain	Fungus-host interaction (plant infection, heme- uptake, biofilm structure)	[77–80]	GT	cVVDGITAIGcTVEDTAcAcTTENLAK
Unknown (KEZ44206.1)		Unknown function		GT	TPTKDELVPAGK
Glucanosyltransferase; Glyco_hydro_72 (KEZ46098.1)	Gelp/Gasp (AfGel2p) GH 72	Cell wall remodeling: elongation of β(1–3) glucan	[30], [71–73]	GT	ILSAGVKPAPSGK
Unknown (KEZ43170.1)		Unknown function		GT	cDQGDGSESATLAYSNcLQK
					cINScPATDVNcLAHcTPVPSPNEDNLNKLHDcAAK
Unknown (SAPIO_CDS2081)		Unknown function		GT	GLTSMQTSIQQNcANVR
Unknown (SAPIO_CDS8694)		Unknown function		GT	SGIcGGEGVVSLYKK
Cerato platanin (SAPIO_CDS5955)		Fungus-host interaction (carbohydrate binding)	[82]	GT	VLTDAPVYNVQYGSGK
Cu/Zn superoxide dismutase (KEZ44265.1)	Cu/Zn SOD	Fungus-host interaction (antioxidant, degradation of superoxide anions)	[65, 66]	RC	TLAHLDPFIR

^*a*^ RC: resting conidia; GT: germ tube.

^*b*^ For more details on the identification of each protein refer to S1 Table and for the MS/MS spectra of proteins identified with a single peptide refer to S2 Fig.

^*c*^ GDSL: is the consensus sequence of Glycine (G), Aspartate (D), Serine (S), and Leucine (L) amino acids around the active site serine

**Table 2:** Sequence analysis of the identified GPI-anchored proteins.

Protein (accession)	Fungal extract^*a*^	S/T^*b*^ (%)	pI	Omega site (ω-5 to ω+5)^*c*^	GRAVY value^*d*^	N- and O- glycosylation
Glucan endo-1,3-β-D-glucosidase (KEZ41172.1)	RC/GT	22.0	4.41	QQAAP**S** AGSSNN	-0.413	7 N + 68 O
CFEM protein (KEZ46909.1)	RC/GT	26.2	4.01	TQGPGNT**G**GAQQS	-0.062	0 N + 40 O
CFEM protein (KEZ46627.1)	RC/GT	17.6	4.37	SSFPT**A** GAGSIA	-0.242	2 N + 24 O
CFEM protein (KEZ44163.1)	RC/GT	22.5	4.24	SAPTSS **G**AAGVV	0.453	1 N + 25 O
GDSL_like lipase (KEZ43142.1)	RC/GT	19	4.45	KAGDQG **S**GAVRV	-0.031	2 N +18 O
Glycine-rich protein (KEZ42341.1)	RC/GT	20.1	6.89	VPADE**S**GARSV	-0.160	5 N + 4 O
Unknown (KEZ44256.1)	RC/GT	21.6	4.25	GSEDS**S**SGDN*K*EGAAASV	-0.205	2 N + 9 O
CRH1_transglycosylase (KEZ42985.1)	GT	23.3	4.69	TGSQD**S** GASLVQ	-0.270	2 N + 31 O
GH17 family protein (SAPIO_CDS10506)	GT	20.3	4.78	ATGADS **S**ASGYT	-0.222	1 N + 32 O
Unknown (KEZ45212.1)	GT	16.1	4.40	SETS*K* G **G**AAELA	-0.035	1 N + 14 O
1,3-β-glucanosyl transferase; gel4 (KEZ46619.1)	GT	13.2	4.70	NKKEDD **S**SAVRF	-0.275	6 N + 13 O
Unknown (KEZ45428.1)	GT	19.8	4.19	GSGDE**G** GAAALA	0.086	3 N + 20 O
CFEM protein (KEZ43031.1)	GT	23.5	3.90	GDDNG**N**GSGTSGAVVN	0.144	3 N + 30 O
Unknown (KEZ44206.1)	GT	19.9	4.69	SPVPT**N** GAARSA	0.090	2 N + 27 O
Glucanosyl transferase Glyco-hydro 72 (KEZ46098.1)	GT	11.6	4.81	STS*K*E**D** AGAFLR	-0.222	3 N + 7 O
Unknown (KEZ43170.1)	GT	25.2	4.20	ATGTG**S** SASATE	-0.112	0 N + 30 O
Unknown (SAPIO_CDS2081)	GT	20.5	4.72	FDIVA**S**PSAHL	-0.243	0 N + 18 O
Unknown (SAPIO_CDS8694)	GT	21	3.40	GPVEV**S** AAGRNT	-1.021	0 N + 120 O
Cerato platanin (SAPIO_CDS5955)	GT	30.2	4.35	VTAAQ**S**AGRRQ	-0.213	0 N + 94 O
Cu/Zn superoxide dismutase (KEZ44265.1)	RC	14.1	4.47	TNLPE**G** SAAVSS	-0.314	4 N + 10 O

^*a*^ RC: resting conidia; GT: germ tube.

^*b*^ S: serine; T: threonine.

^*c*^ Bold: best predicted ω site. Underlined: alternative ω site (second best). Italic: basic amino acids.

^*d*^ GRAVY index > 0: hydrophobic; GRAVY index < 0: hydrophilic.

Analysis of *S*. *apiospermum* proteome also revealed the presence of 100 gene sequences coding for GPI-anchored proteins (S1 Fig), which meant that 20% of GPI-anchored proteins were extracted in our conditions.

## Discussion

Germination and invasiveness are tightly interwound in fungal infections since the presence of hyphae in tissue sections represents a major indicator of fungal burden [40–42]. In filamentous fungi, the conidia-to-hyphae transition is accompanied by cell wall modifications to allow adaptation to new environments. As a matter of fact, in *A*. *fumigatus*, the cell wall of resting conidia is composed of three layers; during germination, the outer hydrophobin/melanin-rich cell wall layer is shed, leading to major changes in the cell wall ultrastructure and physical properties [39, 43–45]. Other major changes during germination of *A*. *fumigatus* include the emergence of β-1,3-glucans to the cell surface leading to the selective recognition of hyphae by Dectin-1 and the decrease in laminin receptors that mediate the adherence to basement membranes [42, 46]. In *C*. *albicans*, Castillo *et al*. [31] also showed that hyphae produced additional GPI-anchored CWPs, Als3 and Rbt1, that are not detected in the cell wall of blastospores. In another study, hyphal induction in *C*. *albicans* was shown to modulate a larger number of GPI-anchored CWPs such as the Als3, Hwp2, Hyr1, Plb5, Sod5, Rhd3, Sod4 and Ywp1 proteins [47].

In this study, we first showed that the cell wall of *S*. *boydii*, which is composed of two layers, undergoes ultrastructural modifications during germination, demonstrated by the transition from a compact electron dense outer cell wall layer in resting conidia and mother cells of germ tubes to a more diffuse and irregular outer layer in hyphae. However, unlike *A*. *fumigatus* that passes by the swelling step during germination, no major changes in the cell size were observed nor any cytoplasm vacuolization, and the outer cell wall layer of the mother cell remained attached to the electron transparent inner layer after germination. Physical properties of the cell surface were also affected by the germination process: conidia and mother cells of germ tubes were more electronegatively charged than hyphae as attested by electrophoretic mobility measurements and cationized ferritin binding. Consequently, the presence of the negatively charged sialic acids was investigated since it could affect the surface charge and was previously correlated to fungal pathogenesis [48] and adhesion of *A*. *fumigatus* conidia to the host basal lamina [49]. In *S*. *boydii*, the neuraminidase treatment did not reduce the binding of cationized ferritin to mother cells suggesting that the surface electronegative charge was not connected to sialic acids. On the contrary, the inhibition of DHN-melanin synthesis in *S*. *boydii* [29] significantly reduced the surface electronegative charge of conidia suggesting an important effect of melanin which is consistent with the reduction of the electronegative charge upon germination and production of hyaline hyphae. Glucuronic acid has also been reported in the cell wall of different fungal species. Therefore involvement of uronic acids in the electronegative charge of *S*. *boydii* cell wall should be investigated. However, to our knowledge, in all studies that have been performed on *Aspergillus fumigatus* [50] or *Fusarium* species [51–53], glucuronic acid was detected from hyphae and not from resting conidia. Therefore it is likely that uronic acids do not contribute in *S*. *boydii* to the higher electronegative charge of conidia compared to hyphae.

In a previous study we demonstrated that the amount of mannose-containing glycoconjugates in the cell wall increased with the maturation of conidia of *S*. *boydii*, but the accessibility of these molecules to Con A was hampered by the accumulation of melanin [29]. As conidia germinate, these mannose-containing glycoconjugates as well as the chitin molecules became unmasked in the hyphal part of *S*. *boydii* germ tubes, thus markedly increasing the binding of Con A and WGA.

The hydrophobic adhesion forces recorded on the conidial surface of *S*. *boydii* (1.8 ± 0.3 nN) were significantly lower than those recorded by Dague *et al*. [39] for *A*. *fumigatus* conidia (3 ± 0.4 nN). *Scedosporium boydii* conidia lacked any peculiar structures formed by rodlet-forming hydrophobins that render the aspergillar surface homogeneously hydrophobic [54]. However, the measurement of CH_3_/CH_3_ (hydrophobic) and OH/OH (hydrophilic) interactions at the surface of conidia and germ tubes showed non-zero values indicating that the whole surface of the conidial and hyphal structures are composed of a mixture of hydrophobic and hydrophilic components. After germination, CH_3_/CH_3_ interactions diminished suggesting a decrease in the content of some hydrophobic glycoproteins. The inhibition of DHN-melanin synthesis in conidia did not affect these hydrophobic adhesion forces, which meant that these interactions were not linked to melanin (data not shown). On the other hand, the OH/OH interactions remained the same before and after germination reflecting no change in some polysaccharide components.

Changes in the cell wall during germination were also illustrated by the analysis of GPI-anchored CWPs. Among the 254 proteins detected from cell wall extracts, only 20 had a GPI-anchor. The presence of non-GPI anchored proteins or atypical proteins was in agreement with some studies [31, 55] where such proteins were consistently found in cell wall extracts even after varying the methodologies of extraction. However in their studies, de Groot *et al*. [35, 56], did not detect atypical proteins.

In yeasts and filamentous fungi, GPI-anchored proteins may be classified into two groups according to their subcellular localization: the plasma membrane proteins (GPI-PMP) and the cell wall proteins (GPI-CWP). Cell wall proteins have abundant *N*- and/or *O*-linked glycosylation sites, a signal peptide [57], and no transmembrane helixes, which was the case for 19 out of the 20 extracted GPI-anchored proteins. Moreover, Pittet and Conzelmann [58] reported that GPI-CWPs had pIs of 4.87 ± 0.22 whereas PMPs had significantly higher pIs of 6.67 ± 0.95. However, a recent study on *Pichia pastoris* showed that proteins in the cell wall or in the plasma membrane did not have different pIs [59]. In our case, all the 19 GPI-anchored proteins had a pI value less than 5.

There is growing evidence that the proximal and distal sequences to the GPI-attachment site (called the ω site), found close to the C-terminus of the protein, exert a major effect on the subcellular localization of a GPI-anchored protein. The presence of a high S/T content (≥ 10%) or stretches of ST upstream the ω-proximal region has been recognized as a marking feature for CWPs [60, 61] since it can override ω-proximal signals like dibasic amino acid signals (arginine (R), histidine (H) and lysine (K)) at ω-1 and ω-2 sites that direct GPI-containing proteins to the plasma membrane [62]. Analysis of the S/T content of GPI-anchored proteins of *A*. *fumigatus* Af293 using proFasta yielded an overall S/T content ≥ 10% for most identified proteins [61]. For the 20 extracted proteins and after the exclusion of N- and C- terminal sequences, the S/T content remained higher than 10%. Moreover, no dibasic amino acids were found at the ω-1 and ω-2 sites (Table 2). Ouyang *et al*. [63] mentioned that a monobasic amino acid can be sufficient for retaining a GPI-anchored protein in the plasma membrane, as was the case for 3 of our extracted proteins, but again the S/T rich regions can override such signal. Add to this, the presence of valine (V), isoleucine (I) or leucine (L) at ω-4 and ω-5 as well as tyrosine (Y) or asparagine (N) at ω-2 was also suggested to act positively for the cell wall localization of proteins according to Hamada *et al*. [64], but these conditions did not apply to our protein sequences neither did they apply to some cell wall proteins in *A*. *fumigatus* like the *Af*Mp1p for example [63]. The ω-proximal signals and cell wall or membrane localization of proteins remain a matter of debate especially that the same GPI-anchored protein might be present in both the cell wall and the membrane compartments [60, 63].

The different families to which these proteins belonged to were also analyzed. Among the 20 identified GPI-anchored proteins identified, one was found only in conidial extracts, whereas 12 were found only in the germ tube extracts and 7 in both extracts. The protein identified only in conidial extracts (KEZ44265.1) carried a Cu/Zn-superoxide dismutase domain. Superoxide dismutases (SOD) are antioxidant enzymes involved in the degradation of superoxide anions. The identified protein in *S*. *boydii* belonged to a particular class of extracellular SODs containing a signal peptide and predicted to have a GPI anchor. These SODs were first described in the opportunistic fungal pathogen *C*. *albicans* that expressed three GPI-anchored SOD1 related proteins: CaSod4p, CaSod5p and CaSod6p [65]. Strains lacking CaSod5p were more susceptible to killing by macrophages and neutrophils and exhibited a decreased virulence in mouse models [65]. Sod5p was suggested to be involved in the degradation of superoxide anions produced by the host cells [66]. In *S*. *apiospermum* genome, seven sequences were predicted to have a superoxide dismutase domain, three of them are Cu/Zn-SODs. Two of the Cu/Zn-SODs have a predicted signal peptide but only one has a predicted GPI-anchor. To be noted, the extracted protein in this study was different from the one previously identified by Lima *et al*. [67] in *S*. *apiospermum* (KEZ41328.1) that did not contain a signal peptide, a transmembrane helix, or a GPI anchor.

Twelve GPI-anchored proteins were only found in germ tube extracts and among these, four proteins belonged to known GPI-anchored protein families implicated in cell wall biosynthesis activities, one to CFEM (Common in Fungal Extracellular Membrane)-domain containing proteins, one to cerato-platanin proteins and the rest had no known functions. The first group of proteins contained a protein similar to Crhp proteins (KEZ42985.1) that are suggested to be involved in the linkage of cell wall polysaccharides β(1–6)glucan and β(1–3)glucan to chitin [68–70]. Crhp proteins are classified in the glycoside hydrolases 16 family (GH16) of the CAZy Database (http://www.cazy.org/). They were originally studied in the yeast *S*. *cerevisiae* but their biochemical function in the fungus remains unknown and single deletions of these genes in *A*. *fumigatus* were not associated to any phenotype changes [71]. Two proteins similar to proteins of the Gel/Gas family were also detected in this study in the germ tube cell wall extract (KEZ466191.1, KEZ46098.1). Proteins of this family were particularly well studied in *A*. *fumigatus*, *Schizosaccharomyces pombe*, *S*. *cerevisiae* and *C*. *albicans* [71]. Gel/Gas proteins belong to the glycoside hydrolase family 72 (GH72) and were shown to perform elongation of β(1–3)glucan chains. To date, only Gel1p, Gel2p and Gel4p in *A*. *fumigatus* have been studied and *Af*Gel2p was shown to be important for cell wall morphogenesis and virulence in a mouse model of invasive aspergillosis while *AfGEL4* was shown to be an essential gene for cell wall remodeling [30, 72, 73]. In *S*. *boydii* germ tube extract, a protein (SAPIO_CDS10506) similar to proteins of the Bgtp/Bglp family was also identified. These proteins belong to the glycoside hydrolase 17 family (GH17) and were studied in *S*. *cerevisiae* and *A*. *fumigatus*. AfBgt2p displays a branching activity in the cell wall by cleaving two residues of a β(1–3)glucan chain and transferring them to another chain of β(1–3)glucan with a β(1–6) linkage *in vitro* [74, 75]. However the single *Afbgt2* mutant strain did not display a differential phenotype with respect to the wild-type strain, thus suggesting that there were other proteins with β(1–3)glucan branching activity in the cell wall [74].

A protein with a predicted CFEM domain was also detected in the hyphal extract. The CFEM domain contains around 60 amino acids, predominantly hydrophobic, and eight cysteine residues with a conserved spacing [76]. These domains are found mainly in GPI-CWPs and most CFEM-containing proteins studied to date are involved in host-pathogen interaction and virulence. In *Magnaporthe grisea*, Pth11p, which contains a CFEM domain, is required for plant infection [77]. Likewise, three proteins with CFEM domain were shown to be involved in cell wall stabilization in *A*. *fumigatus*, but they did not play a role in cell wall morphogenesis or as virulence factors [78]. In *C*. *albicans* proteins containing CFEM domains were suggested to play a role in heme uptake and biofilm structure [79, 80].

In agreement with a previous proteomic study on *S*. *boydii* secreted proteins [81], a cerato-platanin protein was also detected here, from the germ tube extract exclusively. Cerato-platanins are poorly studied proteins, they are thought to be carbohydrate binding virulence factors involved in fungal-plant interactions [82]. They were shown to self-assemble at hydrophobic/hydrophilic interfaces and form protein layers. Under AFM, they were shown to form branched structures or large agglomerates characterized by a disordered assembly of protruding segments [83]. In contrast to the resting conidia, examining the surface of hyphal parts of the germ tubes in *S*. *boydii* under AFM showed the presence of structures of no specific orientation assembled in an isotropic manner on the surface (results not shown). The identification of a cerato-platanin protein in our hyphal extract presents a possible explanation for such structures, but further investigations are needed to confirm such link.

We identified seven proteins present in both conidial and germ tube extracts of which three had no known function or domain and three had CFEM domains. The remaining protein was similar to AfBgt2p (KEZ41172.1) and shared 31.4% homology with its *S*. *boydii* paralogue detected in germ tube extracts mentioned earlier (SAPIO_CDS10506). Both proteins (KEZ41172.1 and SAPIO_CDS10506) contained the two conserved glutamic acid residues of the catalytic site described for proteins of GH17 family [84].

Finally, no hydrophobins could be identified in our extracts using the hydrophobin conserved eight-cystein pattern in proFasta although two genes encoding putative hydrophobins were found in the fungal genome. In *A*. *fumigatus*, Dague *et al* [54] showed that the presence of RodAp hydrophobin accounted for the high hydrophobic adhesions (CH_3_/CH_3_ interactions) measured on the conidial surface. RodAp is a moderately hydrophobic protein with a GRAVY value of 0.245 (*A*. *fumigatus* Af293, protein accession P41746.2) and a suggested GPI-anchor [85]. After analyzing the GRAVY values of the extracted GPI-anchored proteins, only one protein in the conidial extract presented a hydrophobic character (GRAVY > 0, Table 2). Interestingly, this protein was twice more hydrophobic than RodAp (GRAVY = 0.453) and had a CFEM domain (KEZ44163.1). CFEM domains, as previously mentioned, have a conserved eight-cysteine pattern that is distinct from that of hydrophobins; they are commonly identified in GPI-anchored cell wall proteins extracts and have predominant hydrophobic amino acid residues in their sequences (32%- 45% of the total amino acids) [76]. The identified CFEM (KEZ44163.1) was present in both conidial and germ tube extracts, but its relative amount with respect to the extracted conidial or hyphal GPI-anchored proteins (calculated after analyzing the average intensity of the strongest peptides) was twice higher in the conidial extract (2.054%) than in the germ tube extract (1.055%). Even though this remains speculative, this may account for the higher cell surface hydrophobicity and CH_3_/CH_3_ interactions observed on the conidial surface and suggests a hypothesis for future investigations on fungi exhibiting high hydrophobic adhesion forces at the conidial surface and no rodlet layer as *S*. *boydii*.

The analysis of the genome of *S*. *apiospermum* also revealed the presence of 100 gene sequences coding for GPI-anchored proteins which is similar to the number identified in other fungal genomes like *A*. *fumigatus* strains Af293 and A1163 which contain 91 and 85 sequences, respectively [61]. Twenty percent of the proteins identified *in silico* were extracted which is similar to results in other studies. If we take *C*. *albicans* for example, in one study [35], 12 GPI-anchored proteins were extracted out of 104 predicted GPI-anchored proteins (11.5%) while in another study [31], 19 GPI-anchored proteins were extracted after using multiple techniques. In *S*. *pombe* and *S*. *cerevisiae*, four and twelve GPI-anchored proteins could be extracted, respectively [86, 87]. The low number of identified GPI-anchored proteins may be related to some technical limitations due to the low abundance of certain proteins, the difficulty of ionizing some peptides, the high *O*-glycosylation that renders trypsin digestion and peptide identification more difficult or to the need of some particular culture conditions for the expression of these proteins as suggested by the comparison of the results from conidial and hyphal extracts [31].

All these results demonstrate that the cell wall in *S*. *boydii* is a highly dynamic structure whose components, when taken together, give the physical, chemical and molecular fungal cell wall imprint. Today, mapping interactions at the surface of microbial cells at high spatial resolution and correlating such information to molecular data is highly valuable to our understanding of the pathogenesis of fungi.

## Supporting Information

S1 Table Characteristics of GPI-anchored proteins identified in conidial and germ tube extracts. (PDF)

S1 Fig Analysis of *S*. *apiospermum* IHEM 14462 proteome sequences in search for putative GPI-anchored proteins. Accession numbers of analyzed sequences are available on GenBank database (*Scedosporium apiospermum* genome accession number JOWA00000000).
(PDF)

S2 Fig MS/MS spectra from proteins identified with a single-peptide from mycelial (a—i) and conidial extracts (j—o). The major fragmentation series (y-carboxy and b-amino) are annotated; the deduced sequence, m/z value and charge state from the precursor ion are indicated.
(PPTX)
