# Supplementary material for: A Multifaceted Study of Scedosporium boydii Cell Wall Changes during Germination and Identification of GPI-Anchored Proteins
Source: PLoS One. 2015 Jun 3;10(6):e0128680. doi: 10.1371/journal.pone.0128680 (PMC4454578; doi:10.1371/journal.pone.0128680)
Supplement: S1 Table — (PDF) [file pone.0128680.s001.pdf]

Table S1. Characteristics of GPI-anchored proteins identified in conidial and/or germ tube extracts

Protein (accession number)	Fungal extract[a]	Sequence	Charge; *m/z* [Da]	Mascot ion score	Modification[b]	Sequence coverage (%)
Glucan endo-1,3-β-D-glucosidase (KEZ41172.1)	RC	AAQGLDGTNGAFNSAR	2 ; 775.37518	95		3.06
	GT	ISPTGIANKEFAGANPDTLVGYIK	3 ; 826.11169	43		9.58
		EFAGANPDTLVGYIK	2 ; 797.91400	32		
		AAQGLDGTNGAFNSAR	2 ; 775.37335	30		
		SQSDFEAEFKAAQGLDGTNGAFNSAR	3 ; 906.75531	29		
CFEM domain (KEZ46909.1)	RC	AGEFGcQSTDVAcLcR	2 ; 915.88647	76	C6, C13 and C15	12.38
		SRDFVYGIR	2 ; 556.79877	43		
	GT	AGEFGcQSTDVAcLcR	2 ; 915.88623	72	C6, C13 and C15	12.38
		SRDFVYGIR	2 ; 556.79712	36		
		DFVYGIR	2 ; 435.23141	24		
CFEM domain (KEZ46627.1)	RC	QGDWYcGcQPDNmSK	2 ; 931.35608	64	C6, C8 and	8.02

					M13	
	GT	QGDWYcGcQPDNMSK	2 ; 923.35590	59	C6 and C8	26.74
		IQGAATNcVIEAcGGAAGALAVITEVQGIcEEALK	3 ; 1182.26111	55	C8, C13 and C30	
CFEM domain (KEZ44163.1)	RC	IPEcANScVTQATSGNK	2 ; 918.92078	24	C4 and C8	9.83
	GT	IPEcANScVTQATSGNK	2 ; 918.91559	67	C4 and C8	15.61
		IAGcNQGDIK	2 ; 538.26495	33	C4	
GDSL_like lipase (KEZ43142.1)	RC	SQKVVLVDFR	2 ; 595.85217	34		2.83
	GT	MAALLFDGINNAASR	2 ; 782.40625	26		4.25
Glycine-rich protein (KEZ42341.1)	RC	GGSSSSSSSSSRPGSPGFAGSGAPR	3 ; 737.67633	37		8.68
	GT	GGSSSSSSSSSRPGSPGFAGSGAPR	3 ; 737.67267	60		8.68
Unknown function (KEZ44256.1)	RC	NTcEALcPGAAK	2 ; 646.29688	62	C3 and C7	6.32
	GT	YYSASLYSFVcQEAFK	2 ; 981.95807	75	C11	14.74
		NTcEALcPGAAK	2 ; 646.29364	31	C3 and C7	
CRH1_transglycosylase (KEZ42985.1)	GT	GAVFSIANEK	2 ; 518.27850	60		13.14
		LGSWVAGR	2 ; 423.23560	37		
		GGKTYPQTPMQVK	2 ; 717.87677	30		

		TYPQTPMQVK	2 ; 596.80658	28		
		DcPADPAIGGDFTVDFTK	2 ; 963.43573	18	C2	
GH17 family protein (SAPIO_CDS10506)	GT	DSNPDNKMQFAITK	2 ; 804.89087	62		10.82
		NAPGKFNAVR	2 ; 537.29724	42		
		AGIGADPSVLVGFIGDYR	2 ; 903.97845	21		
Unknown function (KEZ45212.1)	GT	AcGATDYDcQcAAQQAISTcYNNcPGDSRK	3 ; 1148.13013	29	C2, C9, C11, C20 and C24	19.23
		AcGATDYDcQcAAQQAISTcYNNcPGDSR	3 ; 1105.43347	25	C2, C9, C11, C20 and C24	
1,3-β-glucanosyltransferase gel4 (KEZ46619.1)	GT	GIAYQQNTGAAGAGVQDAK	2 ; 910.45422	101		6.00
		FFYENGTQFYIK	2 ; 778.87817	21		
Unknown function (KEZ45428.1)	GT	QGLLGVVANAEDGVLYAcSQVK	2 ; 1146.09692	47	C18	9.28
Unknown function (KEZ43031.1)	GT	cVVDGITAIGcTVEDTAcAcTTENLAK	2 ; 1465.16699	44	C1, C11, C18 and C20	13.50
Unknown function (KEZ44206.1)	GT	TPTKDELVPAGK	2 ; 628.34967	48		5.43
Glucanosyltransferase; Glyco_hydro_72 (KEZ46098.1)	GT	ILSAGVKPAPSGK	2 ; 612.87158	33		2.74

Unknown function (KEZ43170.1)	GT	cDQGDGSESATLAYSNcLQK	2 ; 1102.46899	89	C1 and C17	30.77
		cINScPATDVNcLAHcTPVPSPNEDNLNKLHDcAAK	5 ; 819.36963	16	C1, C5, C12, C16 and C33	
Unknown function (SAPIO_CDS2081)	GT	GLTSMQTSIQQNcANVR	2 ; 954.45905	56	C13	7.11
Unknown function (SAPIO_CDS8694)	GT	SGIcGGEGVVSLYKK	2 ; 777.40729	43	C14	2.35
Cerato platanin (SAPIO_CDS5955)	GT	VLTDAPVYNVQYGSGK	2 ; 855.94086	76		3.86
Cu/Zn superoxide dismutase (KEZ44265.1)	RC	TLAHLDPFIR	2 ; 591.83844	23		3.79

[a] RC: resting conidia; GT: germ tube.

[b] For amino acid modifications, C «number » corresponds to carbamidomethyl modification at the indicated amino acid number, and M «number » to oxidation modification at the indicated amino acid number.

For proteins identified with a single peptide refer to the MS/MS spectra in **S2 Fig**.
