# Supplementary material for: A Multifaceted Study of Scedosporium boydii Cell Wall Changes during Germination and Identification of GPI-Anchored Proteins
Source: PLoS One. 2015 Jun 3;10(6):e0128680. doi: 10.1371/journal.pone.0128680 (PMC4454578; doi:10.1371/journal.pone.0128680)
Supplement: S1 Fig — Accession numbers of analyzed sequences are available on GenBank database (Scedosporium apiospermum genome accession number JOWA00000000). (PDF) [file pone.0128680.s002.pdf]

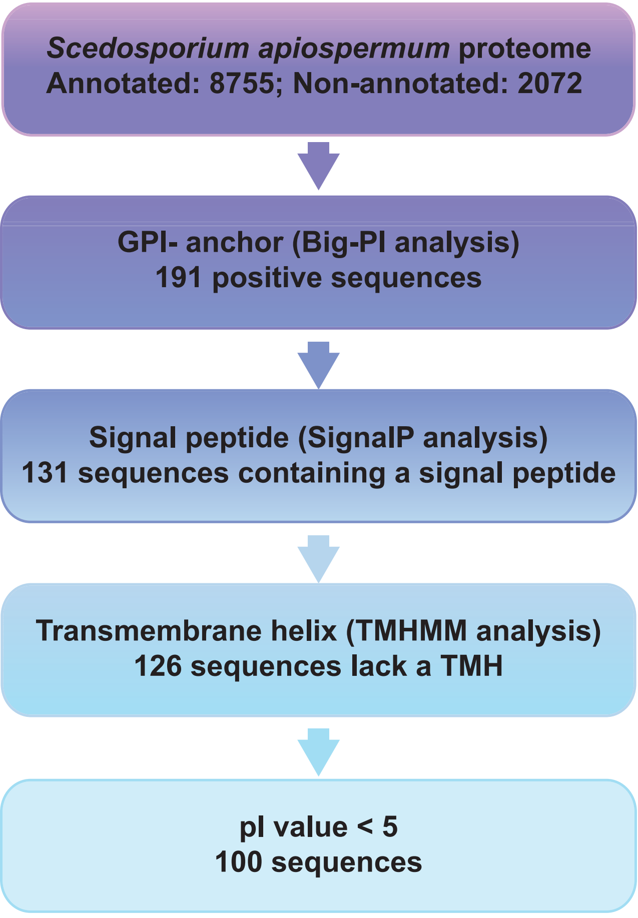
Scedosporium apiospermum proteome
Annotated: 8755; Non-annotated: 2072
GPI- anchor (Big-PI analysis)
191 positive sequences
Signal peptide (SignalP analysis)
131 sequences containing a signal peptide
Transmembrane helix (TMHMM analysis)
126 sequences lack a TMH
pI value < 5
100 sequences
