# Supplementary material for: A Multifaceted Study of Scedosporium boydii Cell Wall Changes during Germination and Identification of GPI-Anchored Proteins
Source: PLoS One. 2015 Jun 3;10(6):e0128680. doi: 10.1371/journal.pone.0128680 (PMC4454578; doi:10.1371/journal.pone.0128680)
Supplement: S2 Fig — The major fragmentation series (y-carboxy and b-amino) are annotated; the deduced sequence, m/z value and charge state from the precursor ion are indicated. (PPTX) [file pone.0128680.s003.pptx]

## Slide 1
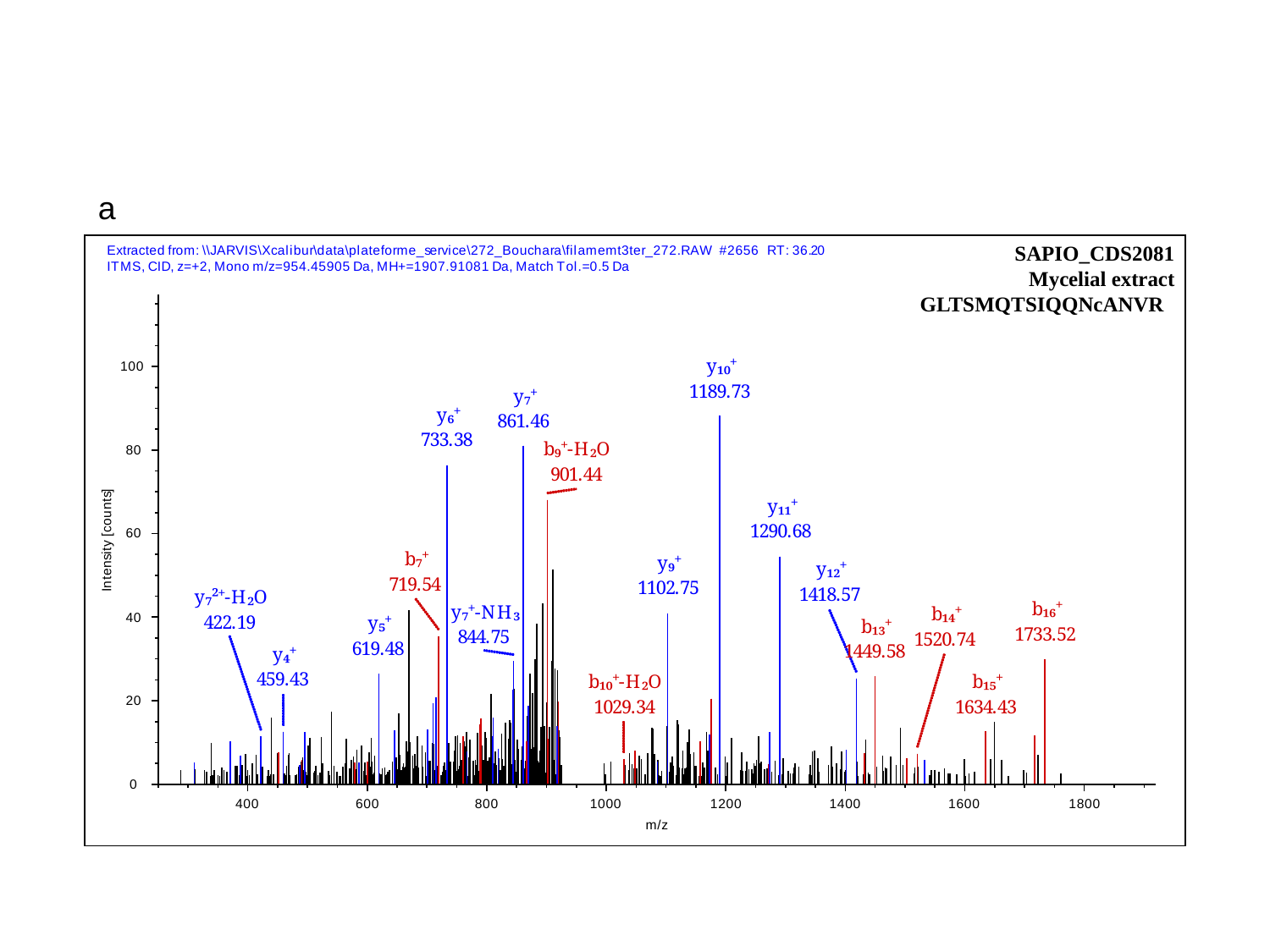

a
SAPIO_CDS2081
Mycelial extract
GLTSMQTSIQQNcANVR

## Slide 2
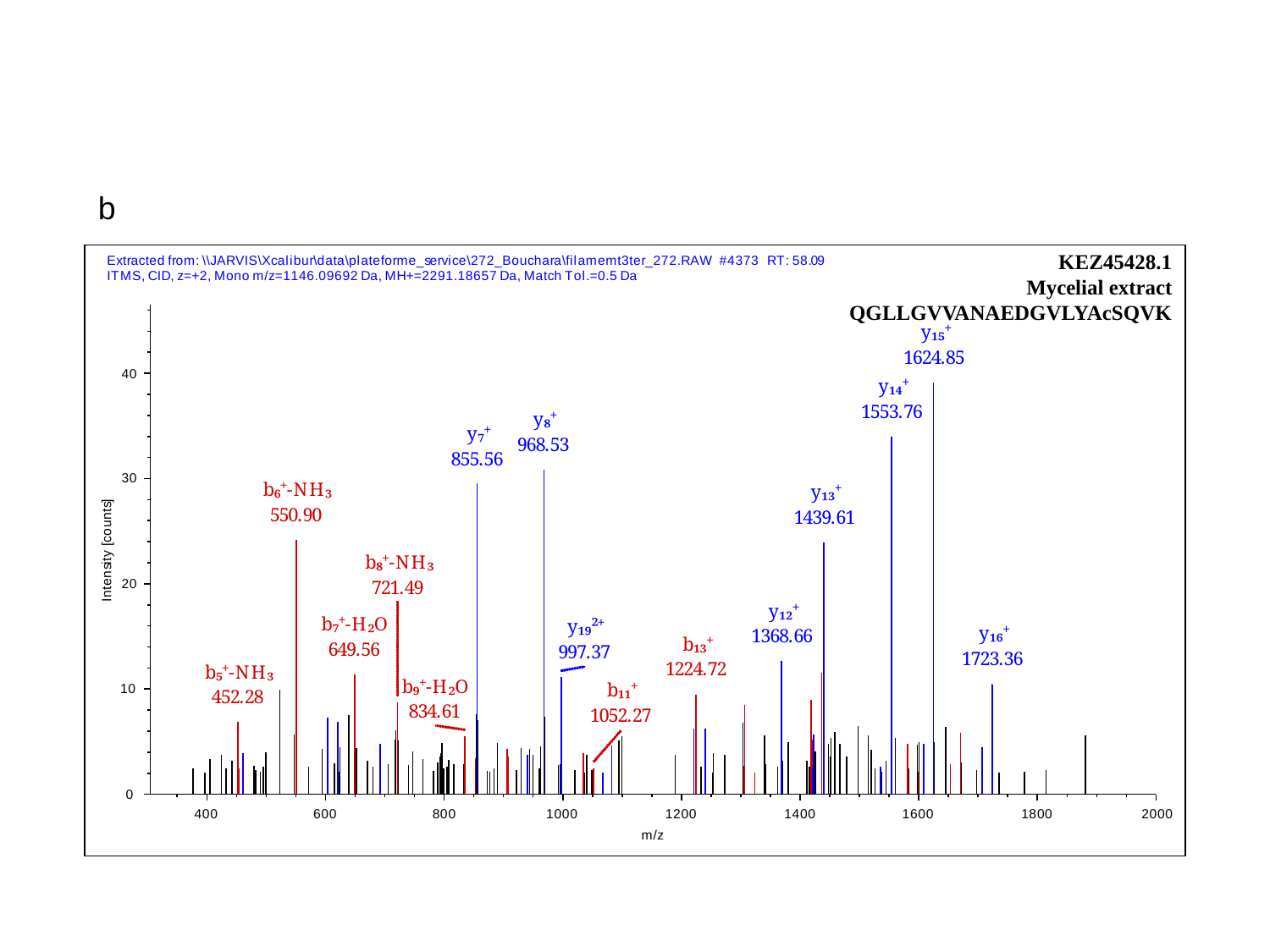

b
KEZ45428.1
Mycelial extract
QGLLGVVANAEDGVLYAcSQVK

## Slide 3
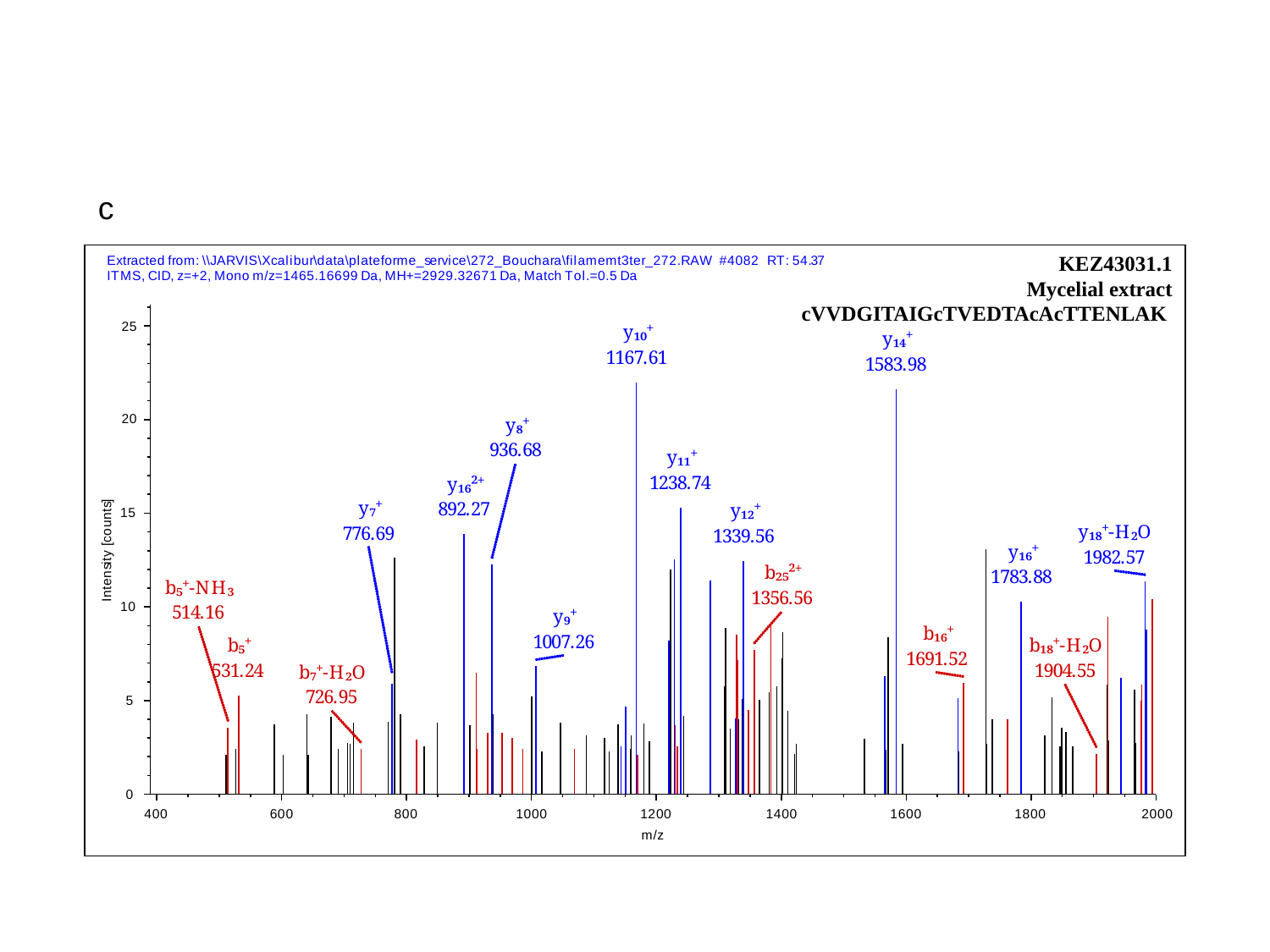

c
KEZ43031.1
Mycelial extract
cVVDGITAIGcTVEDTAcAcTTENLAK

## Slide 4
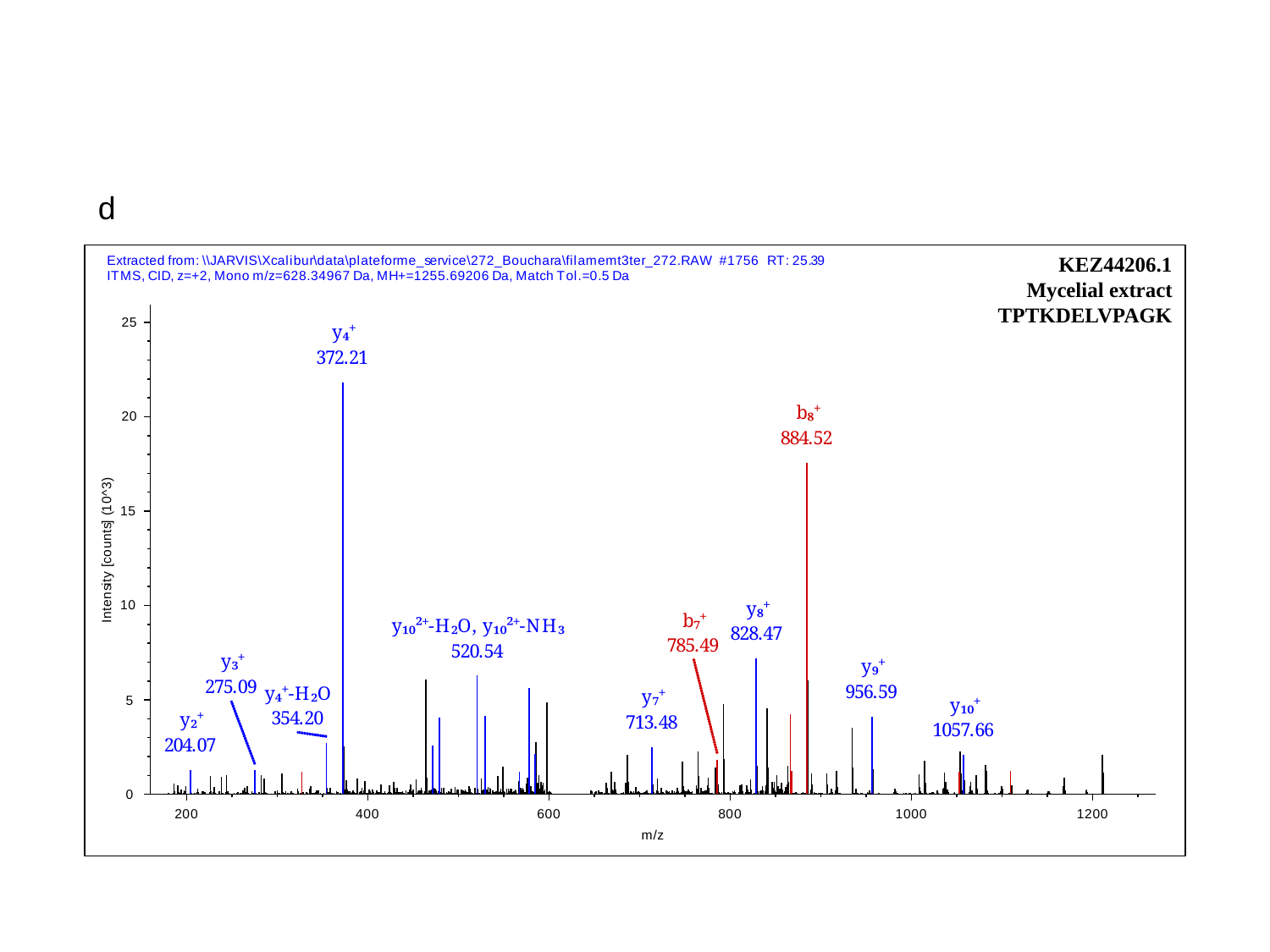

d
KEZ44206.1
Mycelial extract
TPTKDELVPAGK

## Slide 5
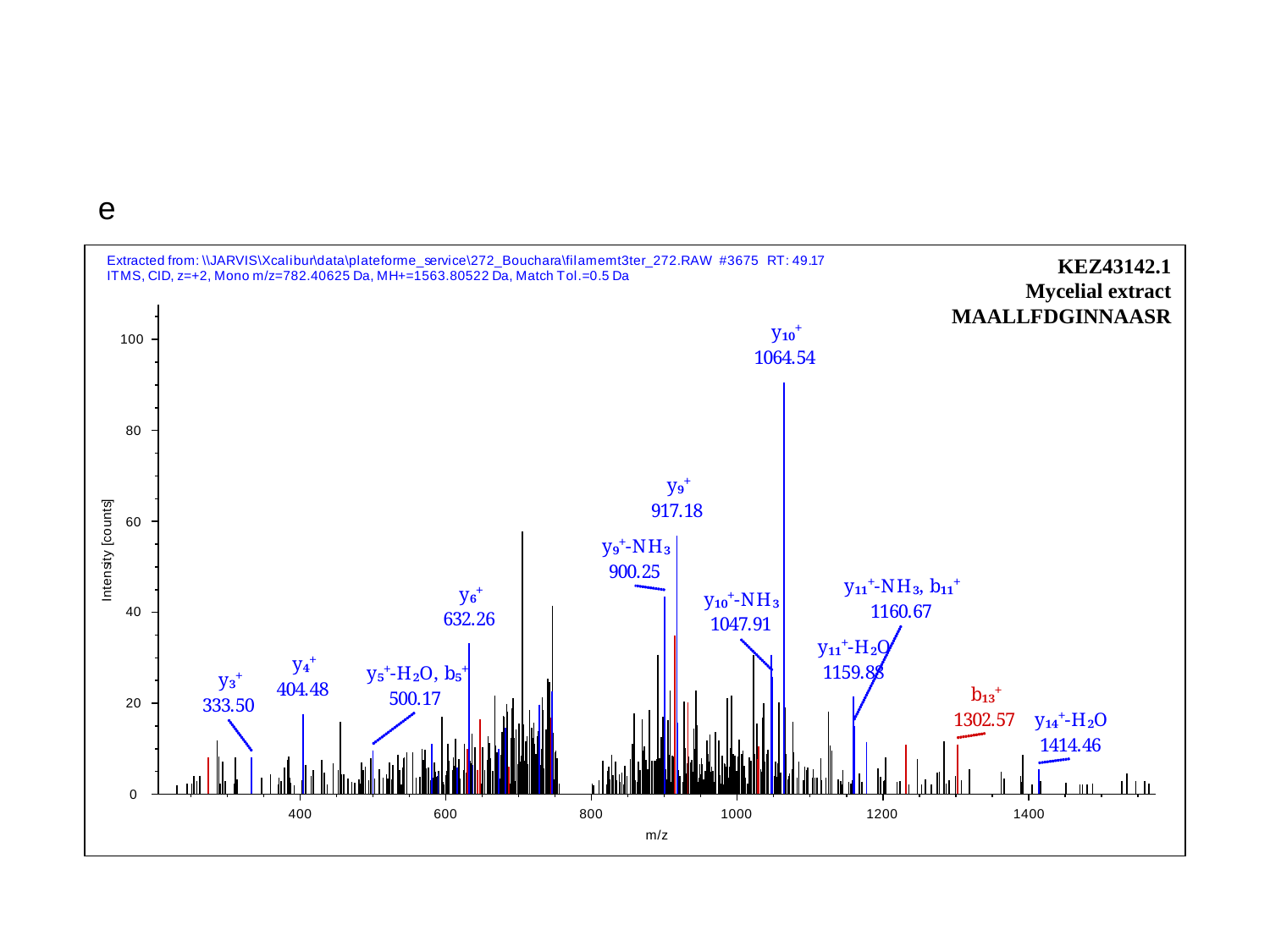

e
KEZ43142.1
Mycelial extract
MAALLFDGINNAASR

## Slide 6
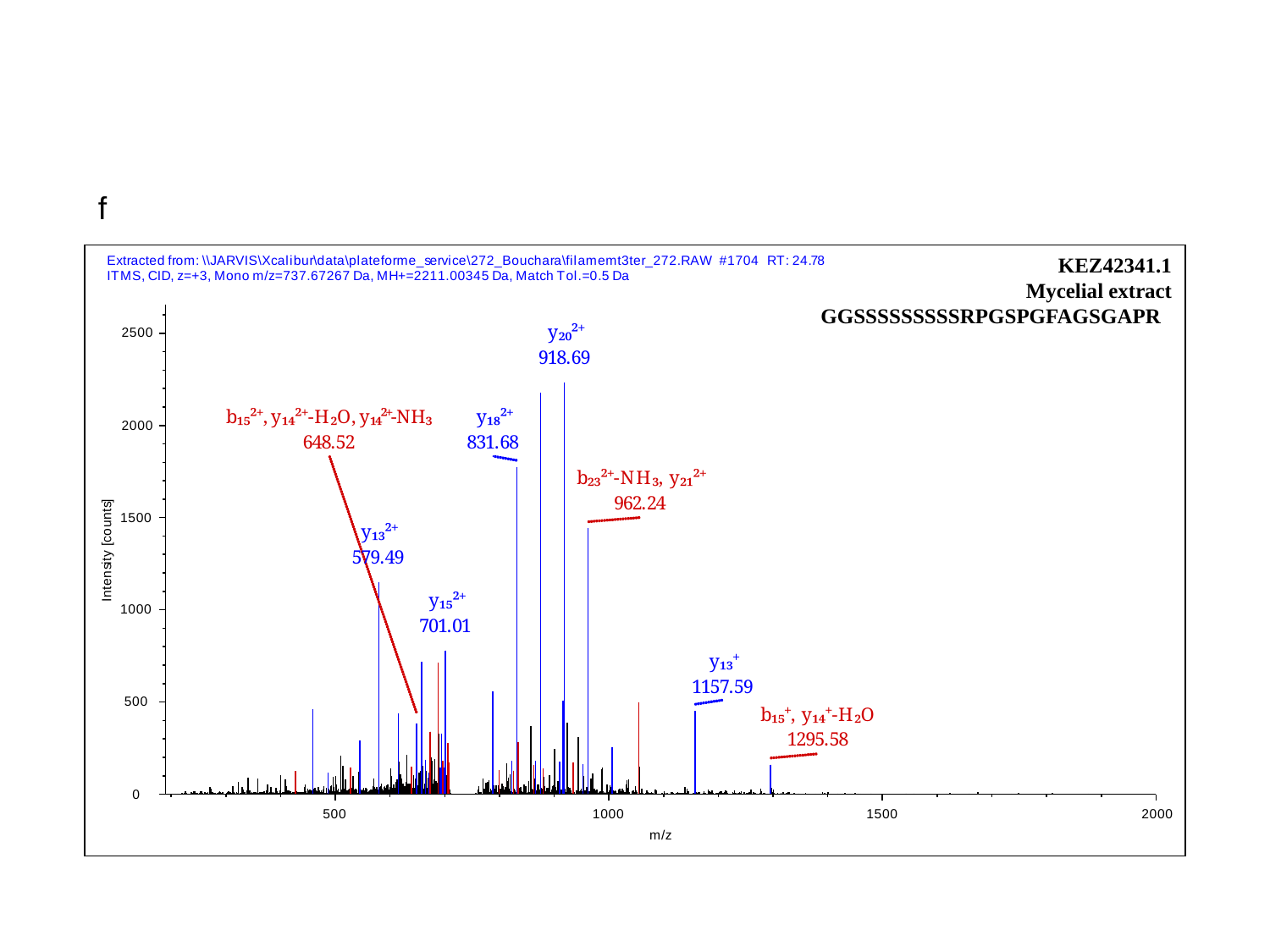

f
KEZ42341.1
Mycelial extract
GGSSSSSSSSSRPGSPGFAGSGAPR

## Slide 7
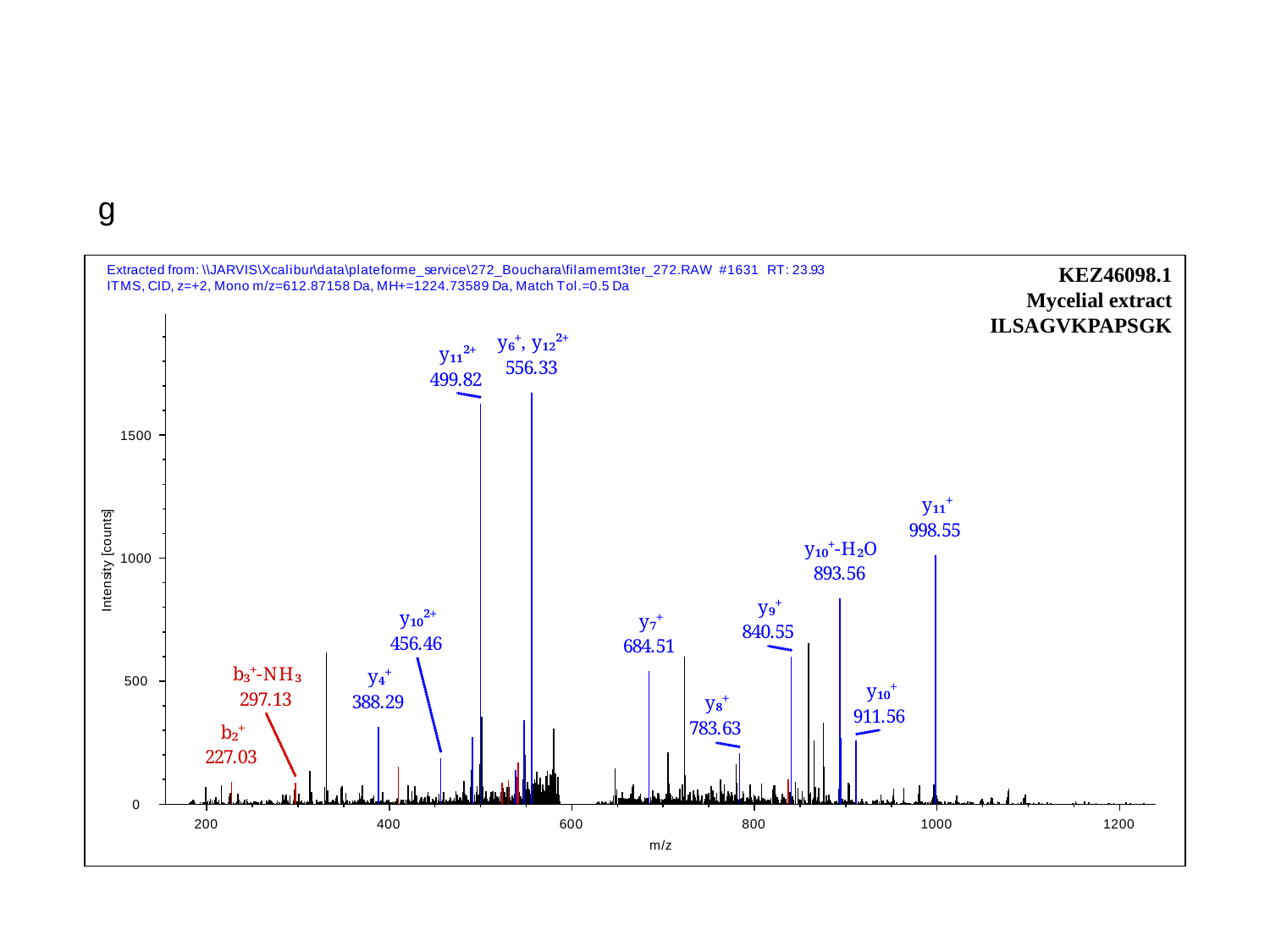

g
KEZ46098.1
Mycelial extract
ILSAGVKPAPSGK

## Slide 8
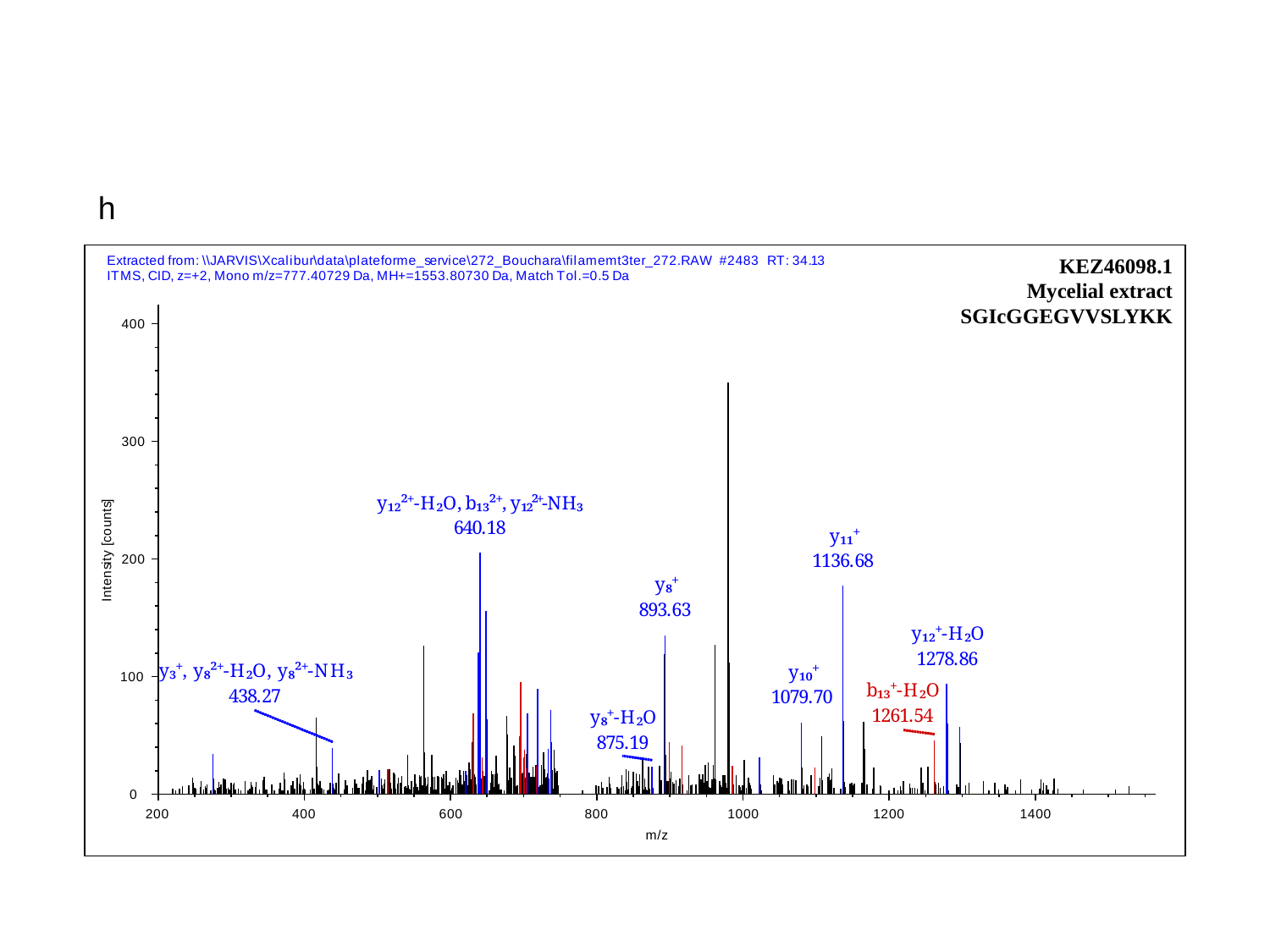

h
KEZ46098.1
Mycelial extract
SGIcGGEGVVSLYKK

## Slide 9
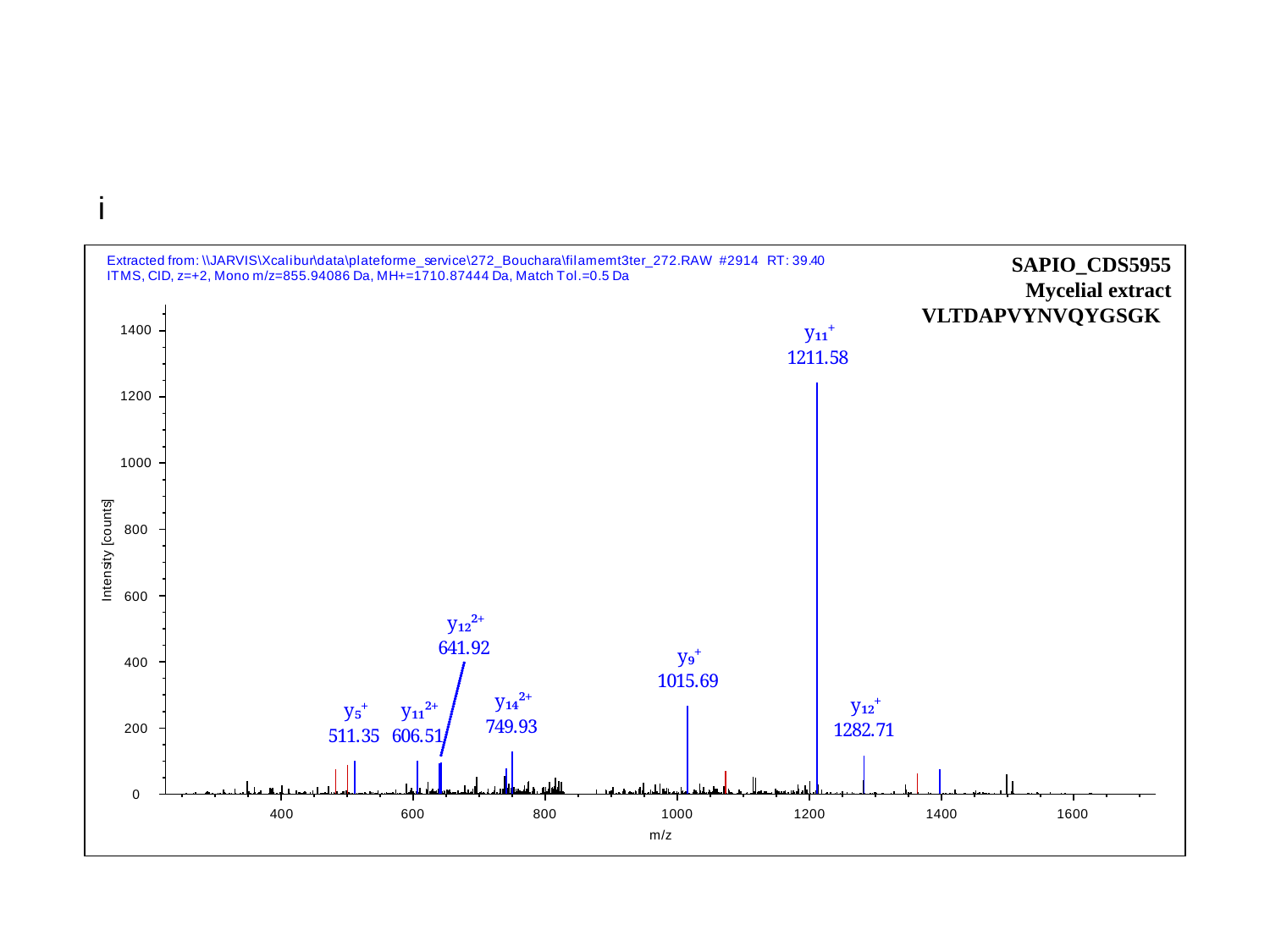

i
SAPIO_CDS5955
Mycelial extract
VLTDAPVYNVQYGSGK

## Slide 10
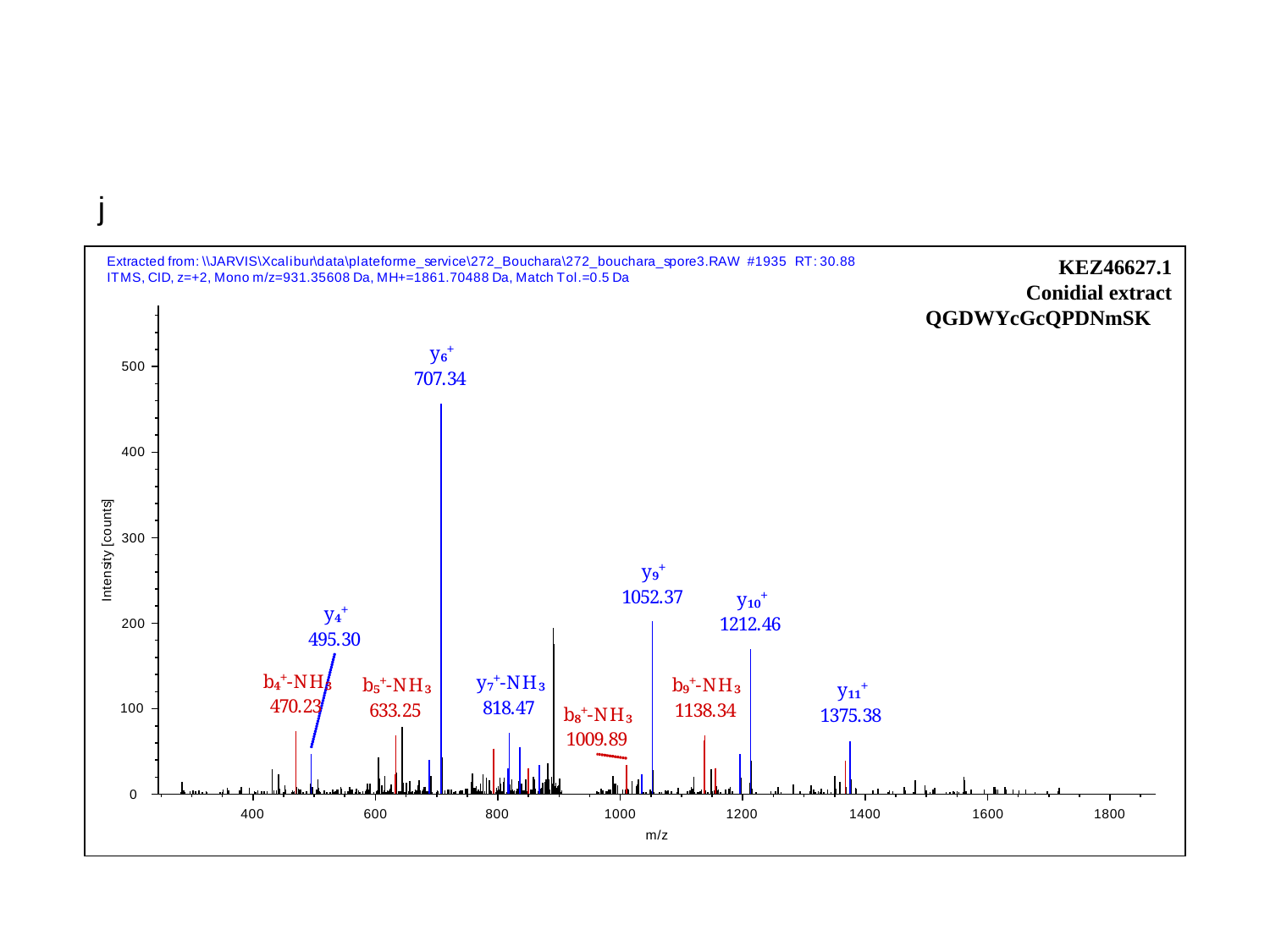

j
KEZ46627.1
Conidial extract
QGDWYcGcQPDNmSK

## Slide 11
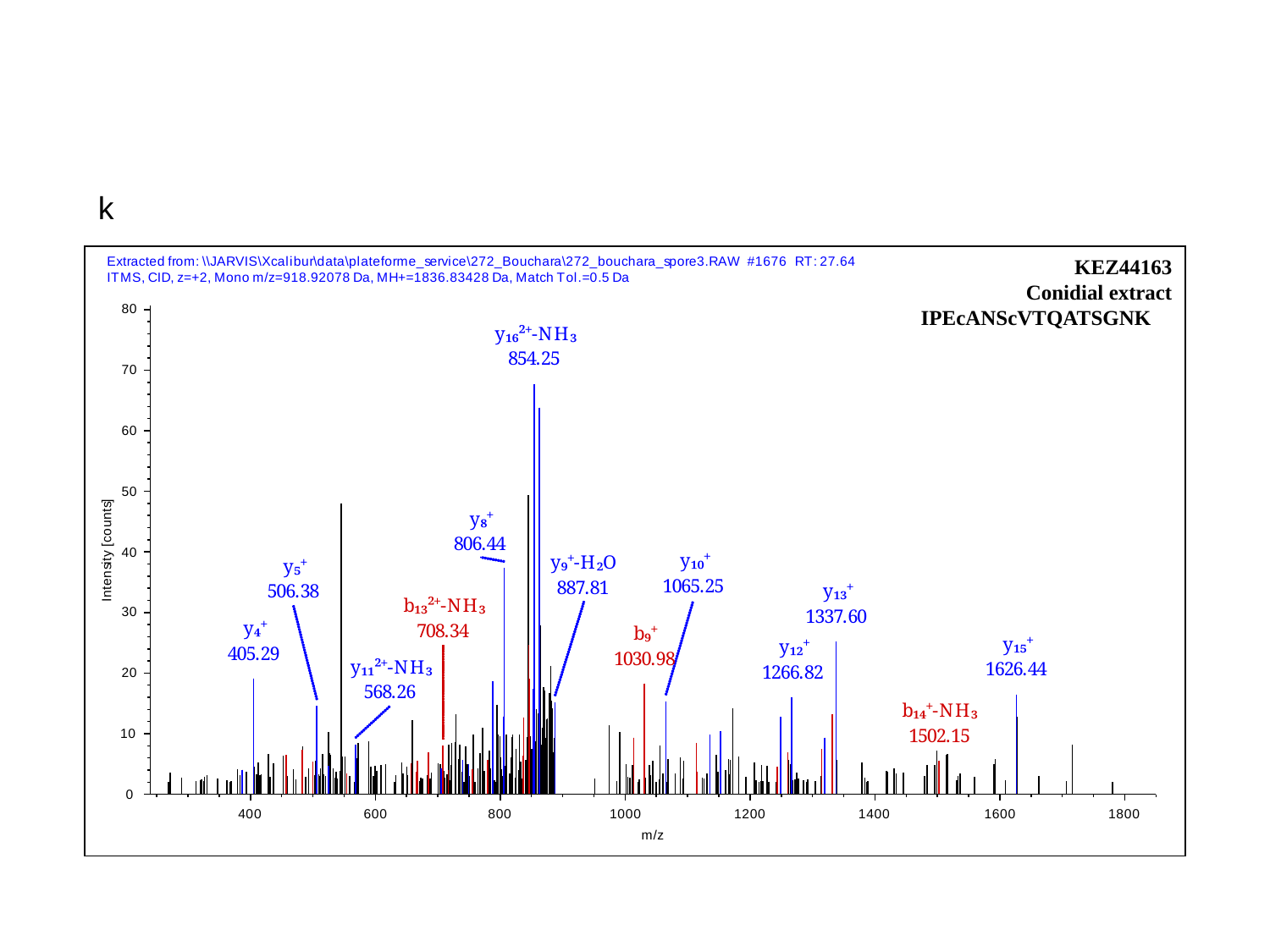

k
KEZ44163
Conidial extract
IPEcANScVTQATSGNK

## Slide 12
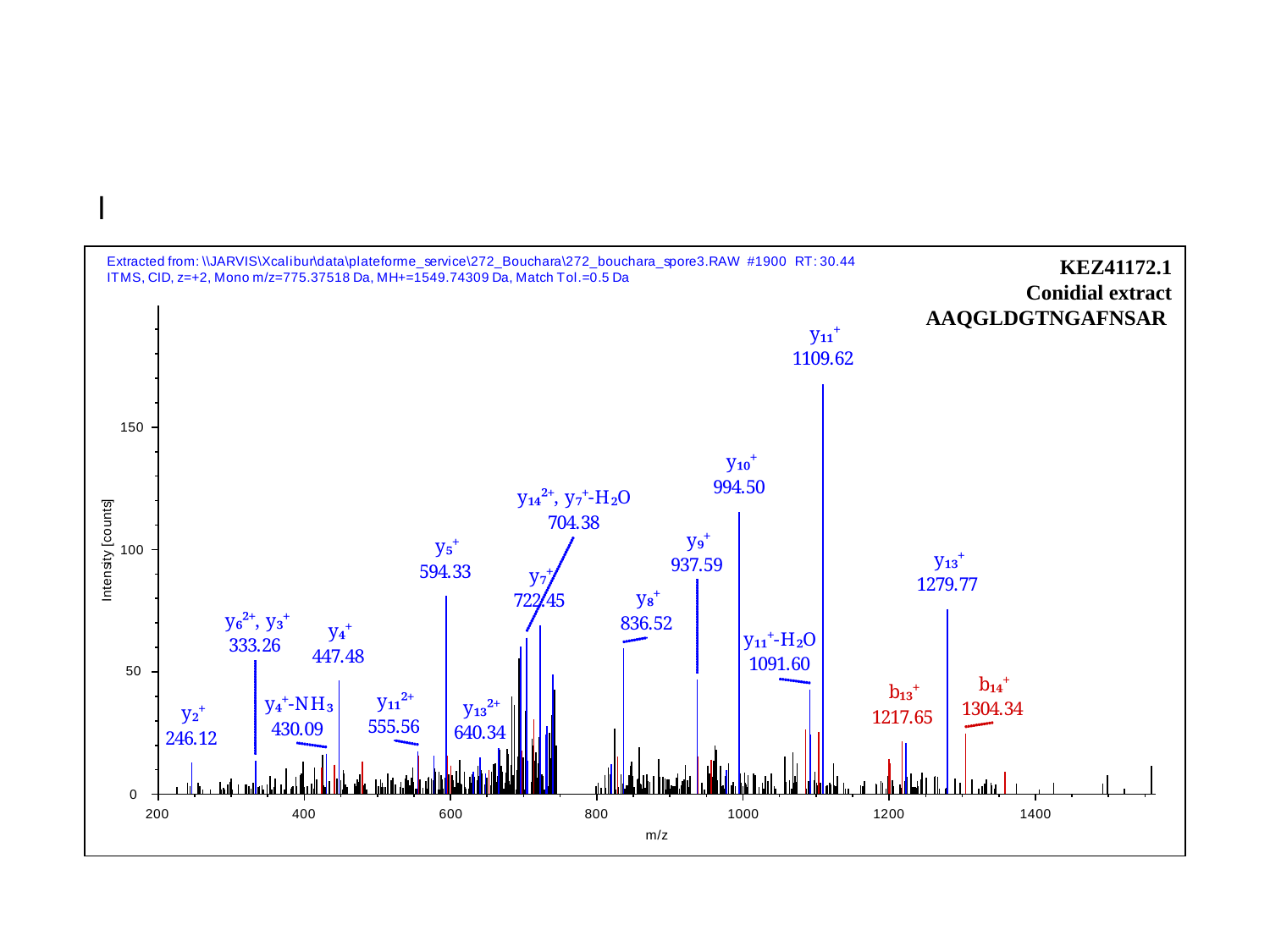

l
KEZ41172.1
Conidial extract
AAQGLDGTNGAFNSAR

## Slide 13
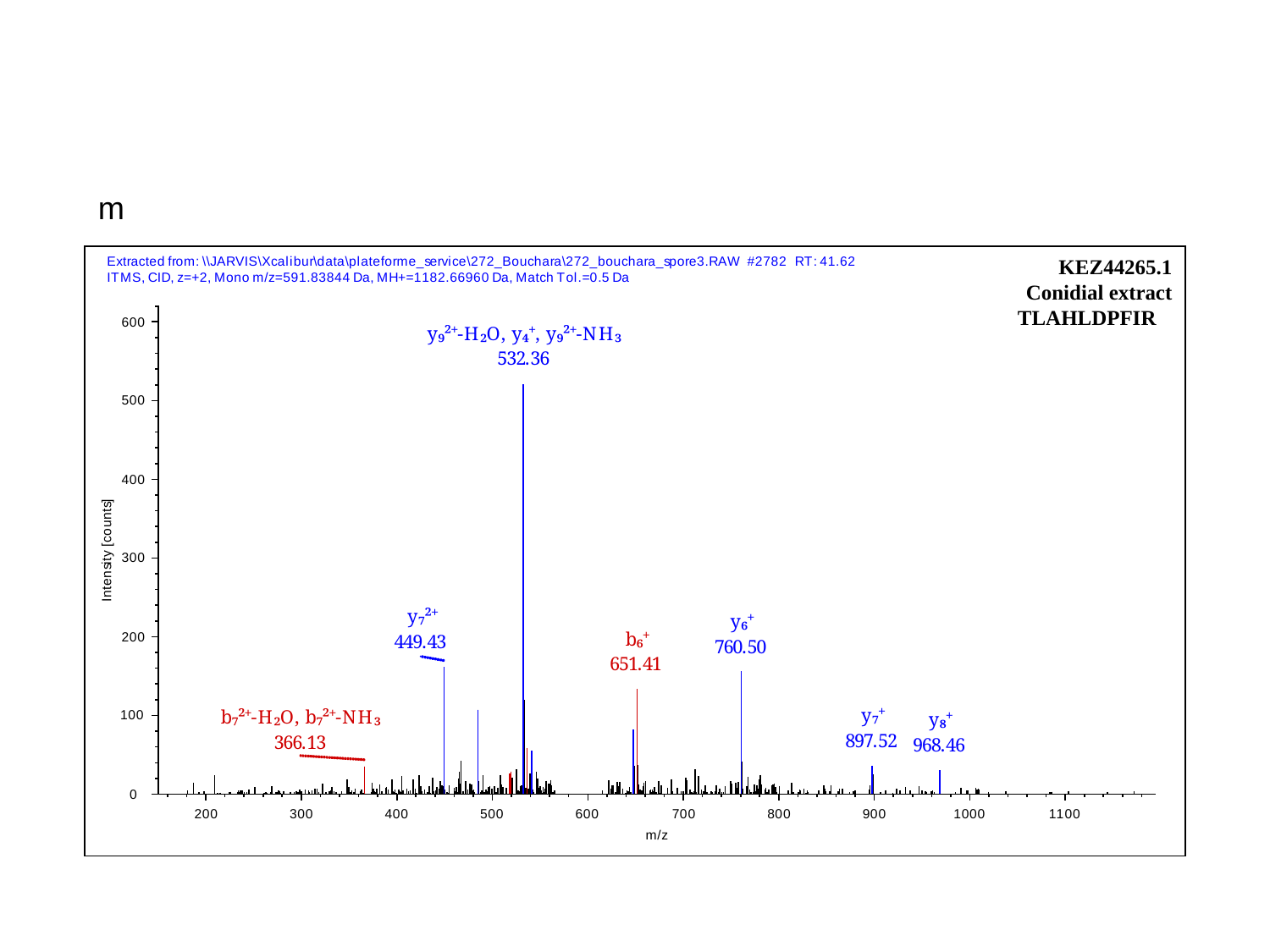

m
KEZ44265.1
Conidial extract
TLAHLDPFIR

## Slide 14
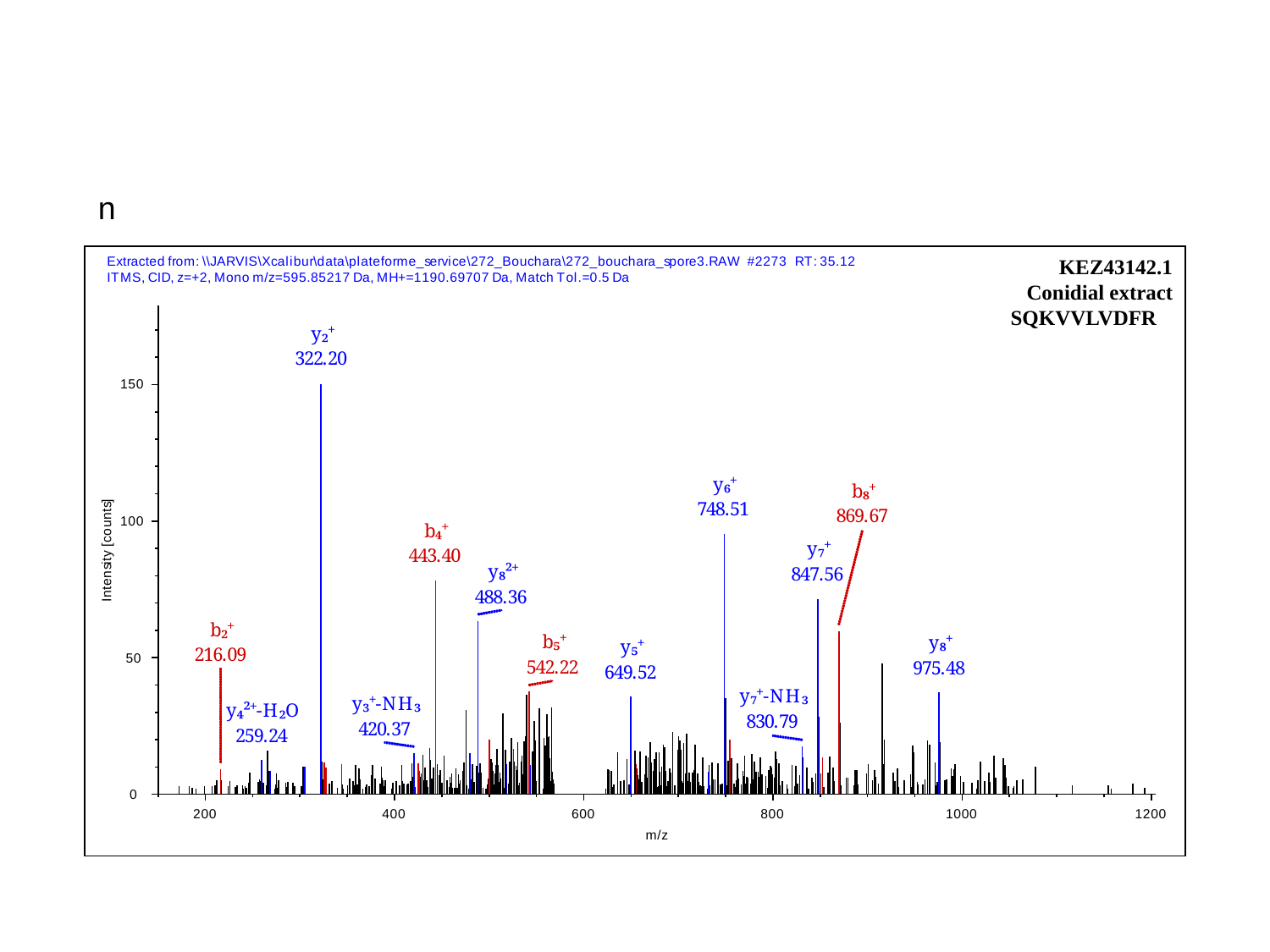

n
KEZ43142.1
Conidial extract
SQKVVLVDFR

## Slide 15
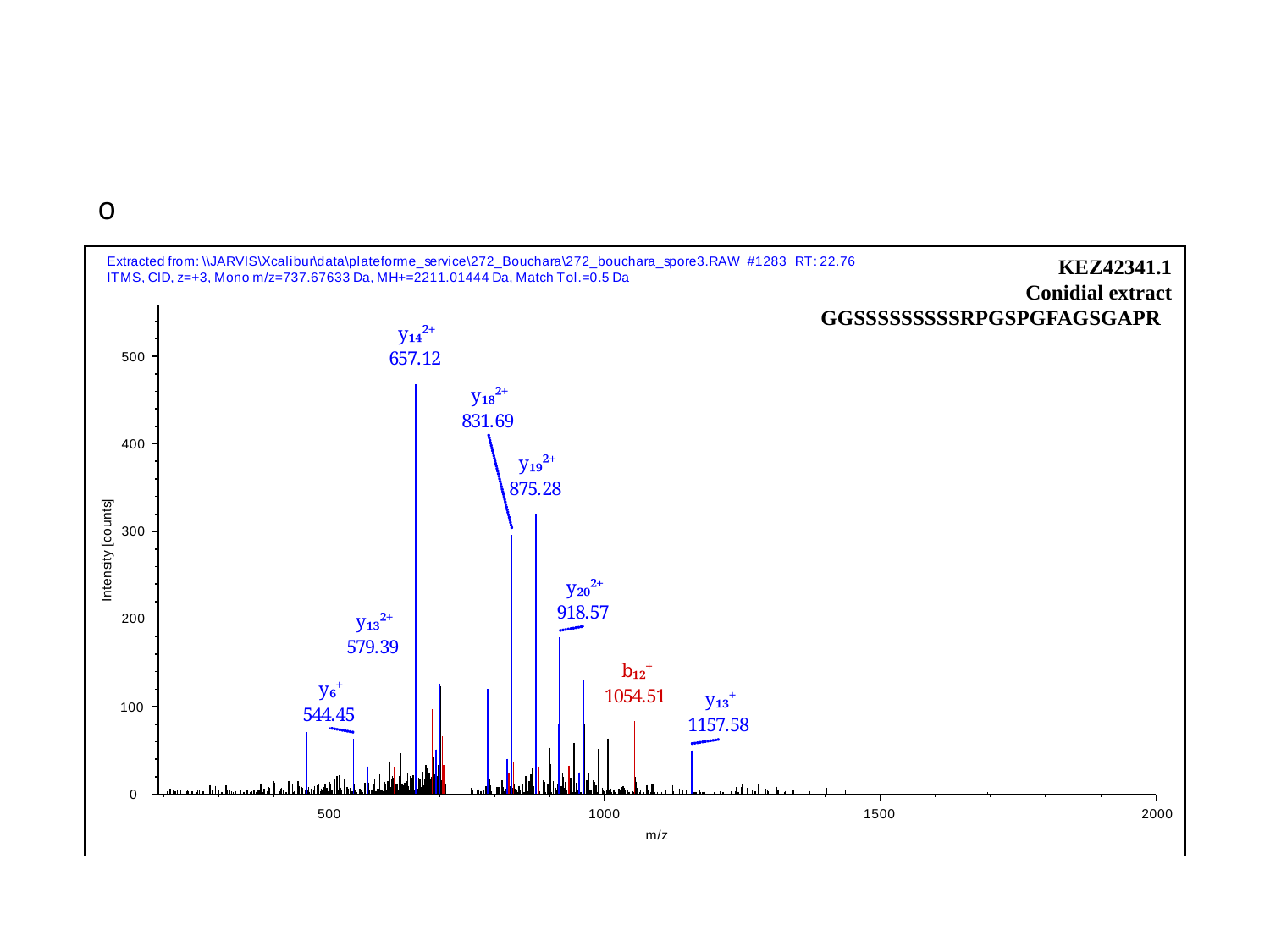

o
KEZ42341.1
Conidial extract
GGSSSSSSSSSRPGSPGFAGSGAPR

## Slide 16
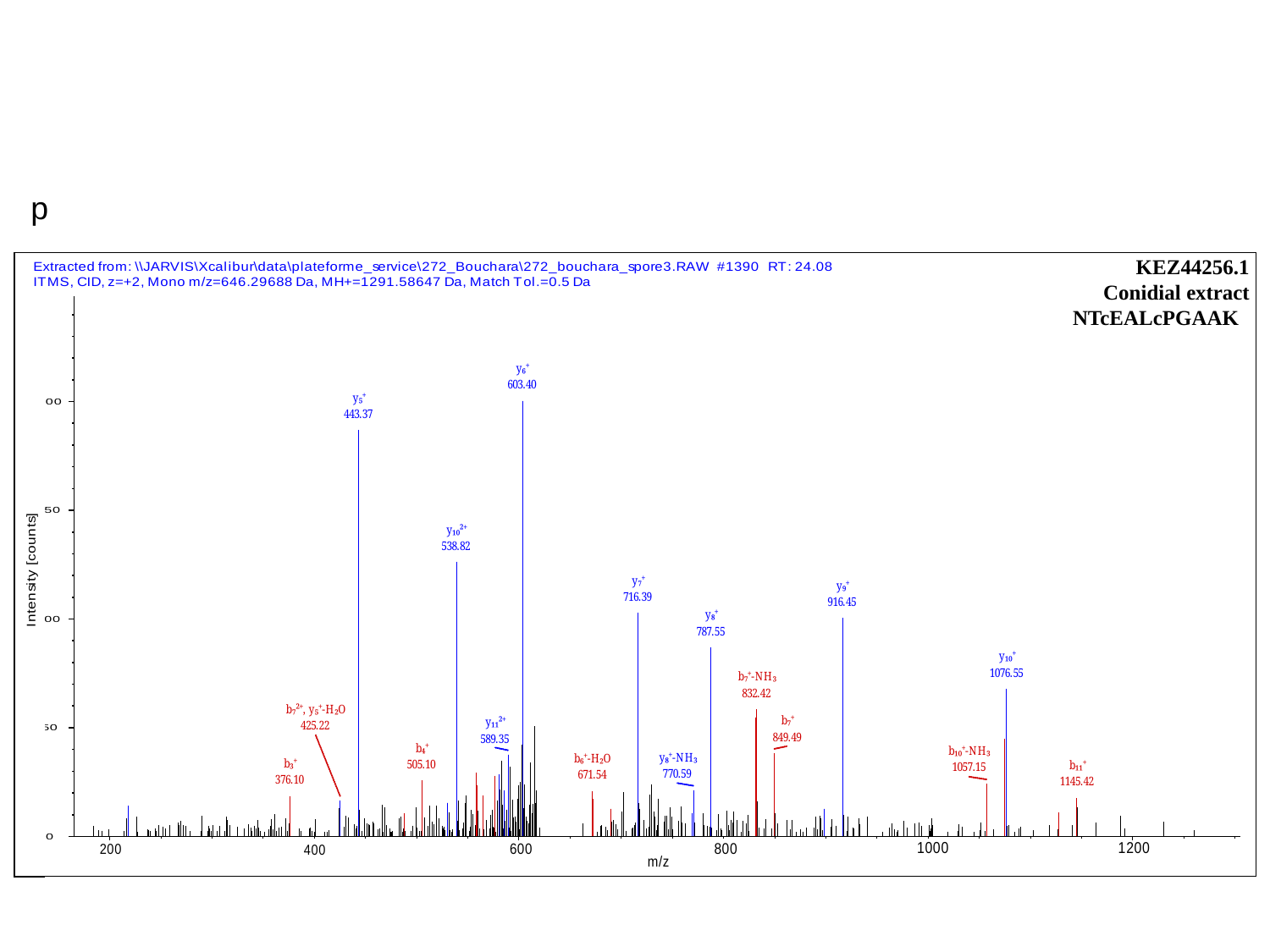

p
KEZ44256.1
Conidial extract
NTcEALcPGAAK

## Slide 17
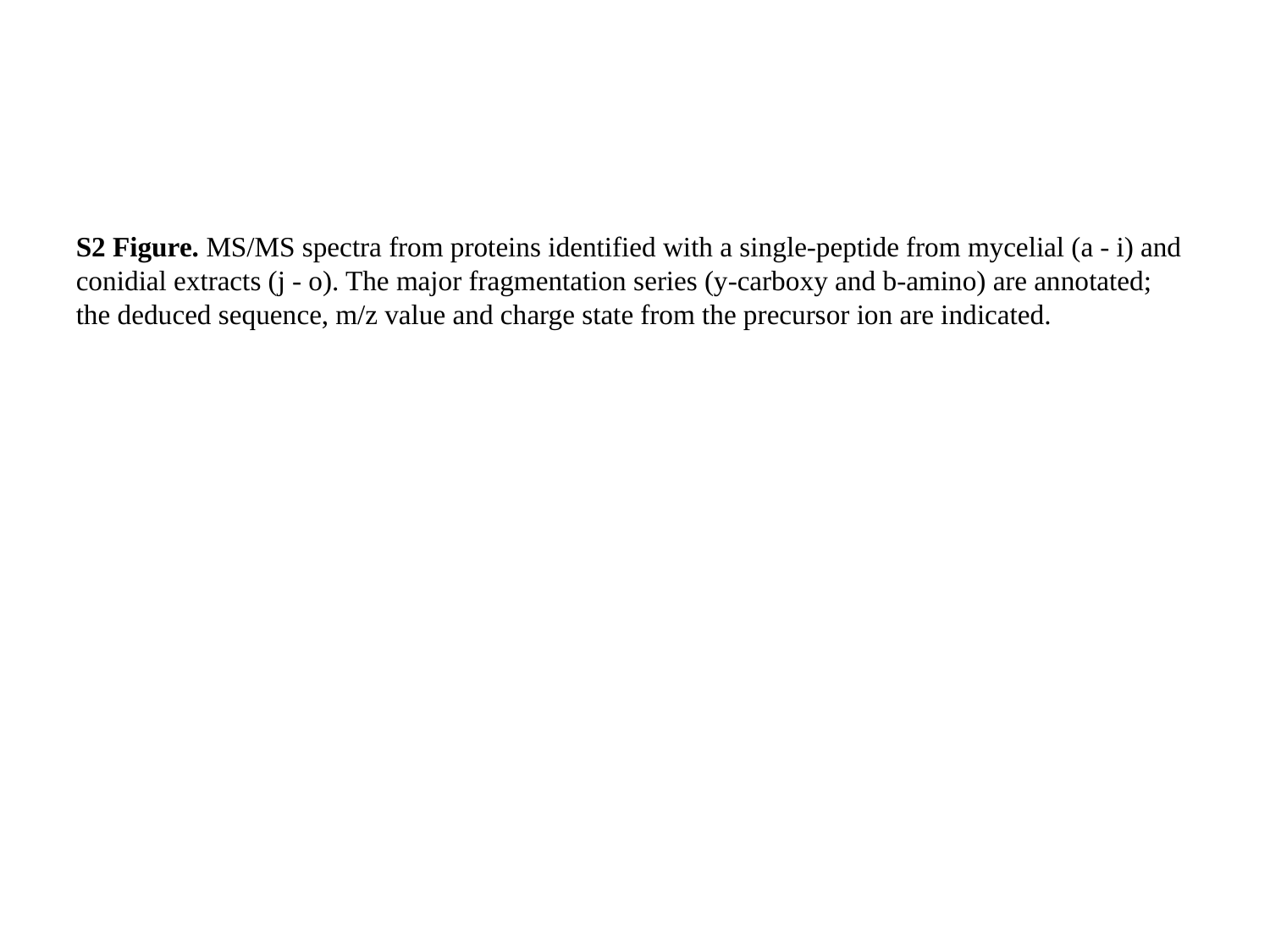

S2 Figure. MS/MS spectra from proteins identified with a single-peptide from mycelial (a - i) and conidial extracts (j - o). The major fragmentation series (y-carboxy and b-amino) are annotated; the deduced sequence, m/z value and charge state from the precursor ion are indicated.
